# ATG12 deficiency results in intracellular glutamine depletion, abrogation of tumor hypoxia and a favorable prognosis in cancer

**DOI:** 10.1080/15548627.2021.2008690

**Published:** 2021-12-14

**Authors:** Tom G. Keulers, Alexander Koch, Marike W. van Gisbergen, Lydie M.O. Barbeau, Marijke I. Zonneveld, Monique C. de Jong, Kim G.M. Savelkouls, Roel G. Wanders, Johan Bussink, Veerle Melotte, Kasper M.A. Rouschop

**Affiliations:** a Department of Radiotherapy, Grow - School for Oncology and Developmental Biology, Maastricht University Medical Centre+, Maastricht, The Netherlands; b Department of Pathology, Grow - School for Oncology and Developmental Biology, Maastricht University Medical Center, Maastricht, Netherlands; c The M-Lab, Department of Precision Medicine, Grow – School for Oncology and Developmental Biology, Maastricht University, Maastricht, The Netherlands; d Department of Radiation Oncology, The Netherlands Cancer Institute, Amsterdam, The Netherlands; e MAASTRO Clinic, Maastricht, Netherlands; f Department of Radiation Oncology, Radboud University Medical Center, Nijmegen, The Netherlands

**Keywords:** Autophagy, cancer, glucose, head and neck cancer, hypoxia, prognosis, radiotherapy glutamine

## Abstract

Hypoxia is a common feature of solid tumors and is associated with increased tumor progression, resistance to therapy and increased metastasis. Hence, tumor hypoxia is a prognostic factor independent of treatment modality. To survive hypoxia, cells activate macroautophagy/autophagy. Paradoxically, in several cancer types, mutations or loss of essential autophagy genes have been reported that are associated with earlier onset of tumor growth. However, to our knowledge, the phenotypic and therapeutic consequences of autophagy deficiency have remained unexplored. In this study, we determined autophagy-defects in head and neck squamous cell carcinoma (HNSCC) and observed that expression of ATG12 (autophagy related 12) was lost in 25%-40% of HNSCC. In line, ATG12 loss is associated with absence of hypoxia, as determined by pimonidazole immunohistochemistry. Hence, ATG12 loss is associated with improved prognosis after therapy in two independent HNSCC cohorts and 7 additional cancer types. In *vivo*, ATG12 targeting resulted in decreased hypoxia tolerance, increased necrosis and sensitivity of the tumor to therapy, but *in vitro* ATG12-deficient cells displayed enhanced survival in nutrient-rich culture medium. Besides oxygen, delivery of glucose was hampered in hypoxic regions *in vivo*, which increases the reliance of cells on other carbon sources (e.g., L-glutamine). We observed decreased intracellular L-glutamine levels in ATG12-deficient cells during hypoxia and increased cell killing after L-glutamine depletion, indicating a central role for ATG12 in maintaining L-glutamine homeostasis. Our results demonstrate that ATG12^low^ tumors represent a phenotypically different subtype that, due to the lowered hypoxia tolerance, display a favorable outcome after therapy.

**Abbreviations:** ARCON:accelerated radiotherapy with carbogen and nicotinamide; ATG: autophagy related; BrdUrd: bromodeoxyuridine; CA9/CAIX: carbonic anhydrase 9; HIF1A/HIF1α: hypoxia inducible factor 1 subunit alpha; HNSCC: head and neck squamous cell carcinoma; HPV: human papilloma virus; HR: hazard ratio; MAP1LC3B/LC3B: microtubule associated protein 1 light chain 3 beta; MEF: mouse embryonic fibroblast; mRNA: messenger ribonucleic acid; PCR: polymerase chain reaction; SLC2A1/GLUT1: solute carrier family 2 member 1; TCGA: the Cancer Genome Atlas; TME: tumor microenvironment; UTR: untranslated region; VEGF: vascular endothelial growth factor

## Introduction

Within the tumor microenvironment (TME), tumor cells can be exposed to a multitude of stressors, including nutrient deprivation and hypoxia. Hypoxia is mainly caused by the (temporary) occlusion of blood vessels (acute hypoxia) or by limited diffusion due to insufficient vascularization (chronic hypoxia). Gradients of oxygenation may vary from normal values (~5% O_2_) to anoxia (~0% O_2_) in relation to the proximity of a functional/perfused blood vessel. From a clinical point of view, means of reducing the hypoxic fraction of tumors is highly desired since low oxygenation of tumors is associated with poor outcome in multiple cancer types [1], independent of treatment modality [2]. In head and neck squamous cell carcinoma (HNSCC), multi-center study indicated that the degree of hypoxia is the most significant factor explaining variability in survival [3]. The observed effect on local control is most likely caused by the resistance of hypoxic cells to both chemo- and radiotherapy. Additionally there appears to be an association between hypoxia and the occurrence of metastasis [4] and it has been proposed that tumor hypoxia contributes to malignancy e.g. through activation of epithelial-to-mesenchymal transition [5–8]. Importantly, a meta-analysis in HNSCC demonstrated therapeutic benefit of hypoxia modification [9]. As tumor hypoxia is the largest determinant in treatment-efficacy variability in survival in head and neck cancers [3] many endogenous markers such as HIF1A/HIF1α, CA9/CAIX, SLC2A1/GLUT1, have been investigated. However, expression of endogenous markers at the site of biopsy does not always comprise the temporo-spatial fluctuations of hypoxia due to its heterogeneous and dynamic characteristics. In addition, these endogenous markers are often hypoxia-responsive targets rather than an indication whether a tumor can intrinsically support survival of a large hypoxic fraction. Biomarkers that causally identify tumor hypoxia are, to our knowledge, still lacking.

Autophagy is a cellular recycling mechanism that targets aged and cytotoxic content, such as aggregated proteins and damaged organelles, for degradation. Hence, during normal physiological and pathological conditions, autophagy is required to maintain cellular homeostasis. Autophagy is particularly active in hypoxic tumor cells and contributes to the survival of hypoxic cancer cells and thereby contributes to regrowth of the tumor and drives tumor cells into a more resistant, invasive and metastatic phenotype. Previously we showed that pharmacological and genetic inhibition of autophagy results in decreased tumor hypoxia and contributes to improved tumor control and treatment efficacy [10].

The process of autophagy commences with an extending double-membrane structure, the phagophore, which (non-)selectively encapsulates the cargo. After closure, the vesicle (the autophagosome) fuses with protease- and hydrolase containing lysosomes, to expose its content to the degrading enzymes. The degradation products amino acids, sugars and fatty acids are effluxed back to the cytoplasm and are used for biogenesis or metabolic purposes [11]. Autophagy is orchestrated by a set of AuTophaGy-related (ATG) proteins, of which most are well-conserved from yeast to higher eukaryotes. At present, more than 40 ATG proteins have been identified and all exert unique functions [12]. Due to its partial integration within the autophagosome and consequent turnover during autophagy, the yeast Atg8 ortholog, MAP1LC3B/LC3B, is frequently used as an autophagy marker [13]. Functionally, LC3B is essential in execution of autophagy as it allows membrane expansion and the fusion of extending membranes of the phagophore to fuse into a vesicle, the autophagosome [12].

Interaction of LC3B with the membrane requires conjugation of LC3B to phosphatidylethanolamine (PE), a process facilitated by the Atg8-family proteins and ATG12 ubiquitin-like conjugation systems [14]. First, pro-LC3B is cleaved by the cysteine protease ATG4 to expose its C-terminal glycine, followed by LC3B transfer to the E1 activating enzyme ATG7. LC3B is then further transferred from the LC3B-ATG7 complex to the conjugating enzyme ATG3 (E2). The ATG12–ATG5-ATG16L1 complex, which serves as the E3-like enzyme recruits the LC3B-ATG7 to the site of expansion. This ATG12–ATG5-ATG16L1 complex is formed by a second ubiquitin-like conjugation system, where ATG7 and ATG10 act as activating (E1) and conjugation enzyme (E2) respectively [15]. The ATG12–ATG5-ATG16L1 complex is essential in expansion of the extending membrane (phagophore) and deletion of *ATG5, ATG16L1* or *ATG12* abolishes autophagosome formation [15–18].

Paradoxical to its role in maintaining hypoxic cell viability, mutations or loss of essential autophagy genes including *ATG2B, ATG9B*, UVRAG (UV radiation resistance associated) and BECN1 (beclin 1) have been reported in gastric, colorectal, renal cell carcinoma, breast- and ovarian cancer [19–21] and are considered to have supported tumorigenesis. Surprisingly, to our knowledge, the phenotypical and therapeutic consequences of autophagy-deficiency have remained unexplored. Current treatment of HNSCC is associated with a high frequency of severe side-effects such as xerostomia. In the past decade, efforts have been made to de-escalate treatment for patients with tumors with favorable prognosis after therapy (HPV+ oropharyngeal), to decrease the therapy associated side-effects. Based on our previous findings, we hypothesized that HNSCC that arise from autophagy deficiency would not be able to support a significant hypoxic fraction and may represent a clinically different subtype with favorable prognosis and may be eligible for dose de-escalation.

Here, we describe for the first time the existence of autophagy-deficient HNSCC. Interestingly, we observed that overall mutation load of autophagy-related genes was very low in HNSCC, but ~25% of the tumors failed to express ATG12. In line, we observed that these tumors lacked tumor hypoxia, displayed improved response to therapy and increased survival of the patients. This effect was confirmed in multiple cancer types of different origin. This is, to our knowledge, the first description of the favorable prognosis of tumors with low autophagy potential. Our findings suggest that (the lack of) ATG12-expression may serve as a useful predictive biomarker for clinical practice to identify cancer patients with a favorable prognosis and warrants future research in autophagy-potential of subclasses in other tumor types.

## Results

### ATG12 expression is lost in a subpopulation of HNSCC

**Figure 1.**
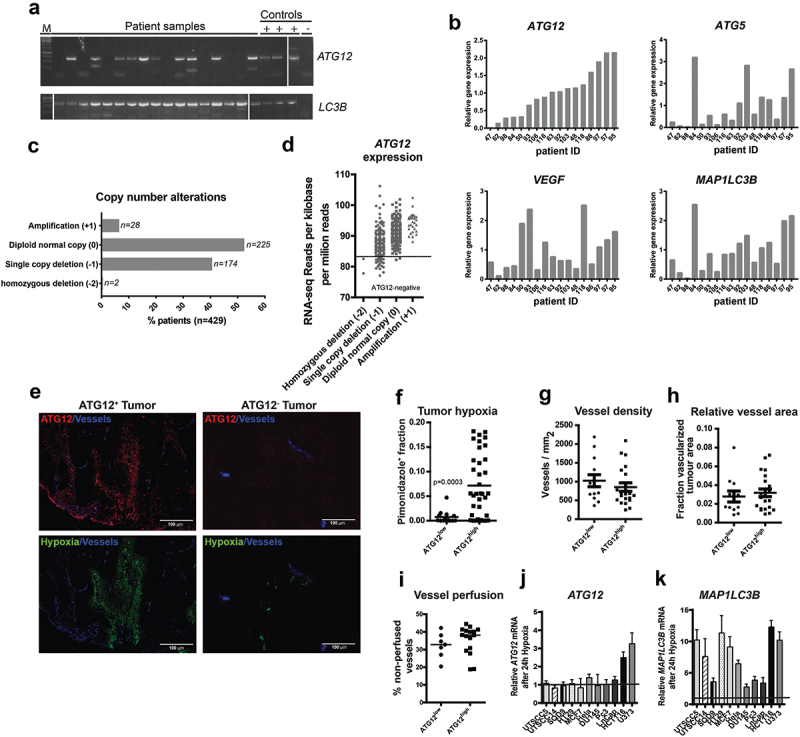
ATG12 expression is lost in a subpopulation of HNSCC. (A) Analysis by conventional PCR displays lack of *ATG12* mRNA transcripts in 7 of 17 patients, whereas *LC3B* is efficiently amplified. (B) Quantitative real-time PCR confirms decreased *ATG12* mRNA abundance in 5 of 17 patients. The hypoxia-regulated proteins *ATG5, LC3B* and *VEGF* do not follow a similar pattern. (C) Within the HPV-negative TCGA HNSCC cohort 176 patients display a single, or homozygous deletion of the *ATG12* gene. (D) Loss in copy number is associated with decreased *ATG12* mRNA presence in the HPV-negative HNSCC TCGA cohort. The range of expression values of patients with homozygous deletions was considered ATG12-negative. (E) Representative immunohistochemical staining of an ATG12-positive (left) and ATG12-negative (right) HNSCC tumor biopsy. ATG12 (Red) and hypoxia (pimonidazole, green). (F) Tumors that lack ATG12 protein expression (ATG12^low^ n = 14) display decreased tumor hypoxia compared to ATG12-proficient tumors (ATG12^high^ n = 37). (G) ATG12^low^ and ATG12^high^ tumors display comparable vessel densities (ATG12^low^ n = 13; ATG12^high^ n = 21). (H) relative vessel areas (vessel size) (ATG12^low^ n = 12; ATG12^high^ n = 22) and (I) comparable vessel perfusion (BrdUrd^+^ in 25-μm radius. ATG12^low^ n = 7; ATG12^high^ n = 16). (J) Real-time quantitative PCR analysis of *ATG12* mRNA expression in 9 cancer cell lines exposed to severe hypoxia (0.02% O_2_) (mean + SEM, n = 3).

Despite the essential role for autophagy in survival of hypoxic tumor cells, mutations or loss of autophagy genes have been described in multiple cancer types [19–21]. To determine if autophagy-defects in HNSCC are existent, RNA was isolated from a panel of 17 HNSCC tumor biopsies of the ARCON trial [22] and screened for mutations and expression in *MAP1LC3B/LC3B, ATG3, ATG5, ATG2B, BECN1, SQSTM1/p62* (sequestosome 1), *ATG12 and ATG9B* (mutations identified in other cancer types or high frequency of nucleotide repeats). No mutations were identified, but we observed lack of expression of *ATG12* mRNA in 7 of 17 patients (Figure 1A) while other autophagy associated mRNAs including *MAP1LC3B/LC3B* (Figure 1A) were well amplified . Next, *ATG12* RNA abundance was measured by qPCR, including the hypoxia-related genes ATG5 and LC3B [23] and VEGF (vascular endothelial growth factor). Although not as binary as in conventional PCR analysis, *ATG12* mRNA abundance was lower in 5 out of 17 patients (29%). *ATG5, LC3B* and *VEGF* expression did not follow a comparable pattern in these patients (Figure 1B). To exclude that reduced amplification was not due to decreased ability of primer recognition, e.g., due to gene truncations or mutations, 3 independent primer pairs targeting the 5ʹUTR, coding sequence and 3ʹ UTR were used. All primer pairs displayed identical patterns (Fig. S1) and thus suggest loss of full transcript expression.

Absence of ATG12 expression in a subfraction of HNSCC was confirmed using the HNSCC cohort of *The Cancer Genome Atlas* (TCGA, cancergenome.nih.gov, n = 479). Of 479 HPV-negative samples within this cohort, 2 (0.4%) tumors displayed homozygous deletion of the *ATG12* gene, 174 (40%) displayed single copy deletion, 225 (52.4%) displayed no genomic changes and 28 (6.5%) displayed gene amplification (Figure 1C) with a dose-response relation between gene- and RNA-expression (Figure 1D). A subset of HPV-negative HNSCC was within the same range as patients with homozygous deletion and could be considered ATG12-deficient. In conclusion, within HNSCC patient populations, large variations in *ATG12* mRNA expression are observed and indicate that a substantial fraction lacks expression of *ATG12*.

### ATG12-deficiency is associated with decreased hypoxia in HNSCC

On the protein level, immunohistochemical analysis revealed an even more striking difference in ATG12 expression and tumors could be classified as either ATG12^positive^ or ATG12^negative^ (Figure 1E). In 14 of the 51 HNSCC tumors with available tissue biopsies [22] (Figure 1F), complete loss of ATG12 in tumor cells was observed. We and others previously showed that autophagy supports survival of hypoxic cells [10]. In this cohort, we observed that expression of ATG12 (or absence) is tumor dependent, applies to the whole tumor and not restricted to hypoxic regions only (Figure 1E). Because ATG12 is required for the general execution of autophagy we characterized the hypoxic fraction and other TME associated parameters (vasculature) of a panel of 51 patients who received the hypoxia marker pimonidazole prior to their biopsy (ARCON trial Nijmegen [22]). Tumor biopsies without or only few (<1%) ATG12-positive cells were grouped as ATG12^low^. ATG12^low^ expressing tumors (n = 14) display no or strongly decreased hypoxic fraction, compared to ATG12 expressing tumors (n = 37) (Figure 1F). In addition to decreased hypoxia tolerance, increased delivery of oxygen will result in lowering reduction of tumor hypoxia. Analysis of the biopsies indicated that tumor vasculature (number and size of vessels) of ATG12^low^ and ATG12^high^ expressing tumors were comparable (Figure 1G and H). Also tumor perfusion, as determined by the surrogate marker, proliferation (bromodeoxyuridine [BrdUrd]-positive signal) in the vicinity (<25-µm radius) of blood vessels [24], did not display differences between ATG12^low^ versus ATG12^high^ groups (Figure 1I). To test if the lack of ATG12 expression is causally related to the absence of oxygen, ATG12 expression of cells exposed to hypoxia was assessed by qPCR. ATG12 mRNA abundance is not regulated by hypoxia in 7 out of 9 cell lines tested (Figure 1J). As observed previously [10], MAP1LC3B mRNA increases after exposure to hypoxia (Figure 1K). These data suggest that the lack of hypoxia is due to loss of ATG12 and not vice versa. This is further supported by the fact that in ATG12^high^ tumors, ATG12 is expressed throughout the tumor and is not restricted to hypoxic areas only (Figure 1E). Together, our data indicate that ATG12 negative tumors either are devoid of tumor hypoxia due to lowered intrinsic hypoxia-resistance.

### Loss of *ATG12* expression is associated with improved prognosis

**Figure 2.**
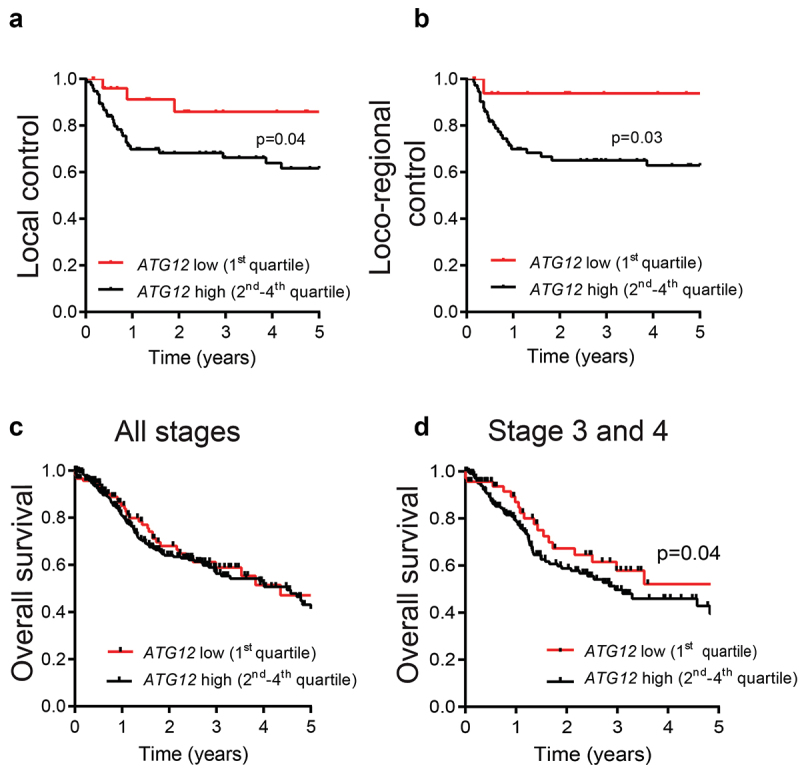
ATG12-deficiency is associated with improved prognosis in late stage HNSCC patients.

Tumor hypoxia is a negative prognostic factor associated with poor treatment outcome [3] and modifying tumor hypoxia results in improved outcome after therapy [25]. Previously we showed that genetic and pharmacological inhibition of autophagy reduces tumor hypoxia and increases efficacy of several treatment modalities including radiotherapy [10,26]. Since ATG12-negative HNSCC tumors display no or strongly decreased hypoxic fraction, we evaluated the effect of ATG12 deficiency on local and loco-regional control in patients that were all treated with (chemo-)radiotherapy. In a cohort of 103 stage 3 and 4 HNSCC patients, *ATG12* mRNA expression was determined by qPCR and divided into quartiles (Table S1). Patients with *ATG12*^low^ (1^st^ quartile) tumors displayed increased local (Figure 2A) and loco-regional control (Figure 2) compared to patients with ATG12^high^ (2^nd^-4^th^ quartile) tumors. The prognostic value of *ATG12* was independent of other covariates such as sex, age, histological site, tumor stage and treatment type (Table S1). Given the independent prognostic value of hypoxia in HNSCC and the lack of tumor hypoxia in ATG12^low^ tumors (Figure 1), these data suggest that *ATG12* acquires its prognostic value through regulating tumor hypoxia.

Kaplan-Meier curves of (A) local control and (B) loco-regional control in HNSCC patients (n = 103) treated in combination with radio- chemotherapy display improved local and locoregional control in *ATG12*^low^ tumors (n = 28 red line) compared to ATG12^high^ tumors (n = 75 black line). ATG12 expression was determined by RT-qPCR and divided in quartiles. Curve comparison was done using the log-rank test, p = 0.04 and 0.03 respectively). (**C**) No difference in overall survival was observed between ATG12^low^ (n = 119) and ATG12^high^ (n = 359) HNSCC patients of the TCGA HNSCC cohort (HPV negative, all stages, n = 479). (**D**) ATG12-expression in HNSCC stage 3 and 4 patients confirms improved prognosis for ATG12^low^ (n = 95) compared to ATG12^high^ patients (n = 279, p = 0.04 (log-rank))

These findings were validated in the TCGA HNSCC patient cohort (HPV-negative, n = 479) (Table S2) of *The Cancer Genome Atlas* (TCGA, cancergenome.nih.gov). Different from the analyzed cohort, outcome data in the TCGA database is limited to overall survival. *ATG12* mRNA expression data and overall survival was extracted from the database and the *ATG12*^low^ group (1^st^ quartile) was compared to *ATG12*^high^ (2^nd^-4^th^ quartile). Interestingly, *ATG12* expression had no prognostic value on overall survival of all patients (stage 1 to 4) (Figure 2C). However, comparable to the data in Figure 2A, in more progressed tumors (stage 3 and 4) *ATG12*^low^ correlated with improved overall survival (Figure 2D). Taken together these data indicate that low *ATG12* mRNA expression is associated with increased local and loco-regional control and thereby contributes to increased overall survival in HNSCC patients. The prognostic value is independent of age, sex, stage and /or tumor site, although a non-significant enrichment for oral cancers in *ATG12*^high^ was observed (Table S2).

**Figure 3.**
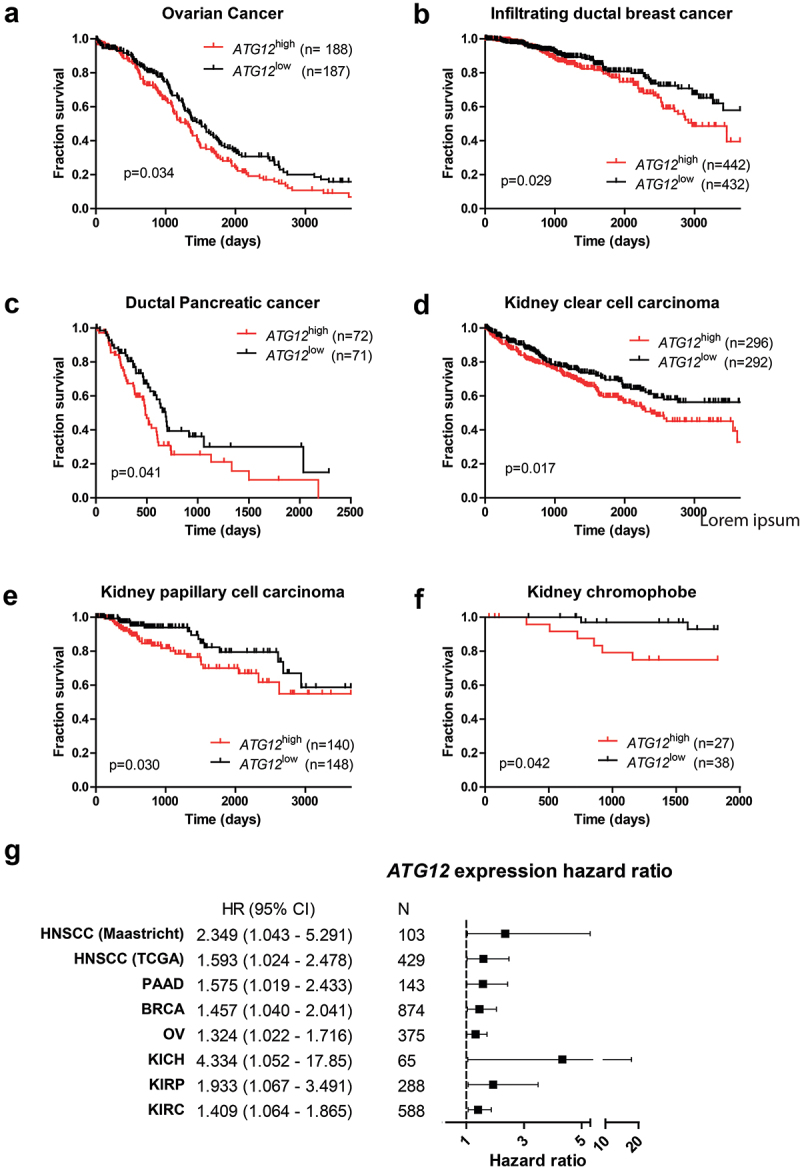
ATG12 expression is associated with prognosis in multiple cancer types. Overall survival of the GDC ATCC (A) ovarian cancer, (B) infiltrating ductal breast cancer, (C) pancreatic cancer, (D) renal clear cell carcinoma, (E) renal papillary cell carcinoma and (F) kidney chromophobe. Patient populations were split on median ATG12 expression. (G) Hazard ratios of the HNSCC cohorts (.Figure 2) and patient populations of Figure 3

Next, we questioned whether the prognostic value of *ATG12* expression was restricted to HNSCC only and mined the TCGA database for additional cancer types. Our prior analysis on the HNSCC cohort indicated that approximately 1 quartile of the HNSCC displayed loss of *ATG12*, justifying splits based on quartiles. However, empirical data to support this approach in other cohorts is unavailable, other patient groups were therefore split based on median expression (Table S3A-F). Interestingly, increased survival associated with low *ATG12*-expression was observed in ovarian cancer (Figure 3A, P = 0.034; HR:1.32 (1.022–1.716)), infiltrating ductal breast cancer (Figure 3B, P = 0.029; HR 1.457 (1.040–2.041)), ductal pancreatic cancer (Figure 3C, P = 0.041; HR:1.575 (1.019–2.433)), renal clear cell carcinoma (Figure 3D, P = 0.017; HR:1.409 (1.064–1.865)), renal papillary cell carcinoma (Figure 3E, P = 0.030; HR 1.933 (1.067–3.491)) and kidney chromophobe cancer (Figure 3F, P = 0.042; HR:4.334 (1.052–17.85)). A summary of the associated hazard ratios and 95% confidence interval of tumor recurrence (HNSCC Figure 2A and B) and death (Figure 2D and Figure 3A-F) in cancer patients with low *ATG12* expressing tumors is plotted in Figure 3G. No direct prognostic value of ATG12 was observed in the TCGA cohorts cervical cancer, bladder cancer, glioblastoma, esophageal cancer, liver cancer and lung cancer. This may be related to low numbers in cohorts, mixed patient populations, inclusion to low grade tumors only, differences in tumor phenotype and use of alternative nutrient resources and/or reliance of hypoxic cells on autophagy for survival.

### Loss of *ATG12* expression contributes to improved tumor control

**Figure 4.**
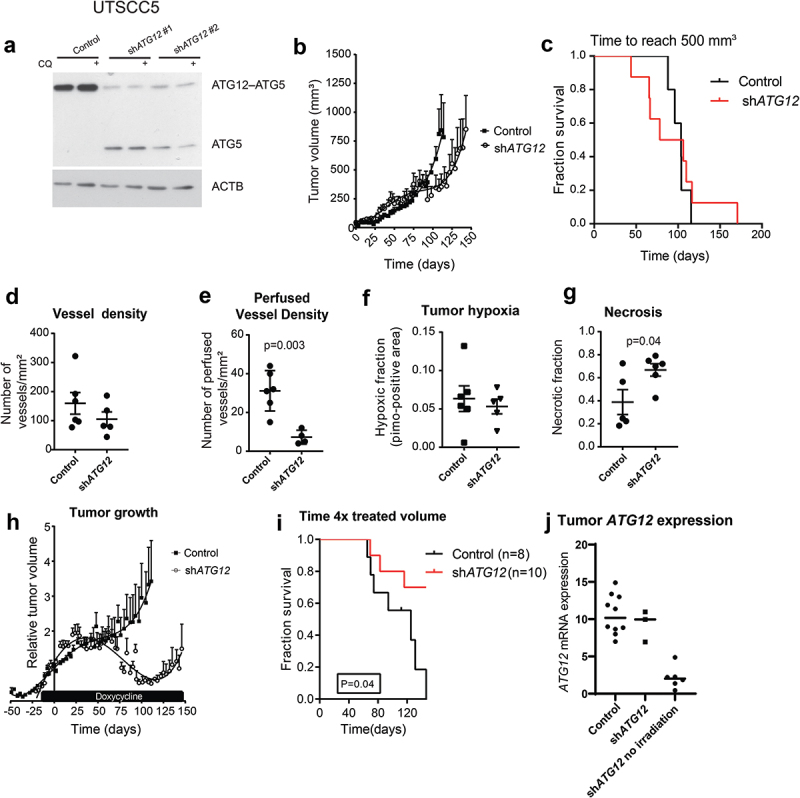
Loss of ATG12 expression contributes to improved tumor control. (A) immunoblot of UTSCC5 cells expressing a doxycycline inducible control shRNA or 2 independent shRNA that target ATG12. Chloroquine (CQ) is used to measure LC3B-II turnover as autophagy activity. (B) Growth curves of UTSCC5 control (n = 5, open circles) and inducible sh*ATG12* #1 (n = 8, filled squares) xenografts. (C) Kaplan-meier curve of tumors reaching 500 mm^3^. (D) Vessel density was determined by 9F1 immunohistochemistry (error bars ±SEM). (E) Perfused vessel density of UTSCC5 xenografts is decreased in ATG12 knockdown xenografts (error bars ±SEM, p = 0.003). (F) Hypoxic fraction was determined using pimonidazole immunohistochemistry (error bars ±SEM). (G) Tumor necrosis of UTSCC5 xenografts was examined morphologically by H&E staining (error bars ±SEM, p = 0.04). (H) Established tumors were irradiated (10 Gy) when reached 150 mm^3^. Doxycycline (5 g/L) was administered via the drinking water 7 days prior to the treatment to induce expressing of the hairpin. In ATG12 knockdown xenografts (open circles, n = 10, error bars ±SEM) tumor regrowth is delayed compared to controls (filled squares, n = 10, error bars ±SEM). (I) Kaplan-meier curve xenografts of tumors reaching 4 x the irradiated volume). (J) human *ATG12* mRNA abundance determined in shSCR (n = 10) and sh*ATG12* (n = 3) tumors that regrew after irradiation. As a reference, knockdown in non-irradiated and size-matched sh*ATG12* tumors (n = 6) is shown (right column).

ATG12 is located on chromosome 5.q, a chromosomal region frequently deleted in head and neck cancer [27]. To determine if the observed effects in patients are causally related to the loss of ATG12-expression and not to the loss of other genes located in the same chromosomal region, we engineered the HNSCC cell line, UTSCC5, with ATG12-inducible (doxycycline) knockdown. Loss in ATG12–ATG5 complex formation and the lack of LC3B-flux, indicated functional loss of ATG12 in these cells after doxycycline exposure (Figure 4A). To monitor *in vivo* effects, these cells were subcutaneously implanted in the flanks of NMRI^nu/nu^ mice. No differences in growth rates were observed (Figure 4B, and C), while knockdown was maintained over time (Fig S2). Similar as in HNSCC patients (Figure 1G) no differences in vessel density after ATG12 knockdown was observed (Figure 4D). Remarkably, the number of perfused vessels is significantly decreased in ATG12 knockdown tumors (Figure 4E). Although reduced perfusion is observed in ATG12-deficient tumors and thus a larger hypoxic fraction would be expected, immunohistochemistry of the exogenous hypoxia marker pimonidazole displayed no changes in the relative fraction of the viable tumor area (Figure 4F). Further analysis indicated that ATG12-deficient tumors displayed an increased necrotic phenotype (Figure 4G). The reduction in the absolute number of hypoxic cells within the tumor, despite reduced perfusion, suggests that ATG12 deficiency results in decreased hypoxia tolerance of cells.

In patients, loss in ATG12 expression contributed to improved outcome after therapy (Figure 2). To test if this loss is the causal factor of this observation, UTSCC5 tumors were established as wild type until they reached ±150mm^3^. Once the tumors reached 150 mm^3^, doxycycline was administered via the drinking water to induce knockdown of ATG12, followed by tumor irradiation (10 Gy single fraction). As hypoxic cells are more resistant to irradiation, regrowth of the tumor after delivery of a single high dose of irradiation is dependent on the number of clonogenic hypoxic cells at the time of irradiation. As expected, tumor irradiation of control tumors resulted in growth delay (Figure 4H). Regrowth, as expressed in times to reach 4x the irradiated volume, was significantly increased in ATG12 knockdown xenografts (Figure 4I). 7 of the 10 ATG12-deficient tumors regressed completely and were still not visible at the time where all control tumors had reached their endpoint (Figure 4I). Analysis of the regrown tumors by human-specific *ATG12* quantitative PCR, revealed that ATG12 expression was regained in the sh*ATG12* tumors that regrew after irradiation (Figure 4J), whereas ATG12-deficient tumors, not treated with irradiation maintained knockdown until the end of the experiment. These results confirm the elevated sensitivity of ATG12-deficient cancers to radiation. These data indicate that, in line with patient data, loss in ATG12 expression contributes to improved response to therapy.

### ATG12 expression determines survival during hypoxia through maintaining intracellular L-glutamine levels

**Figure 5.**
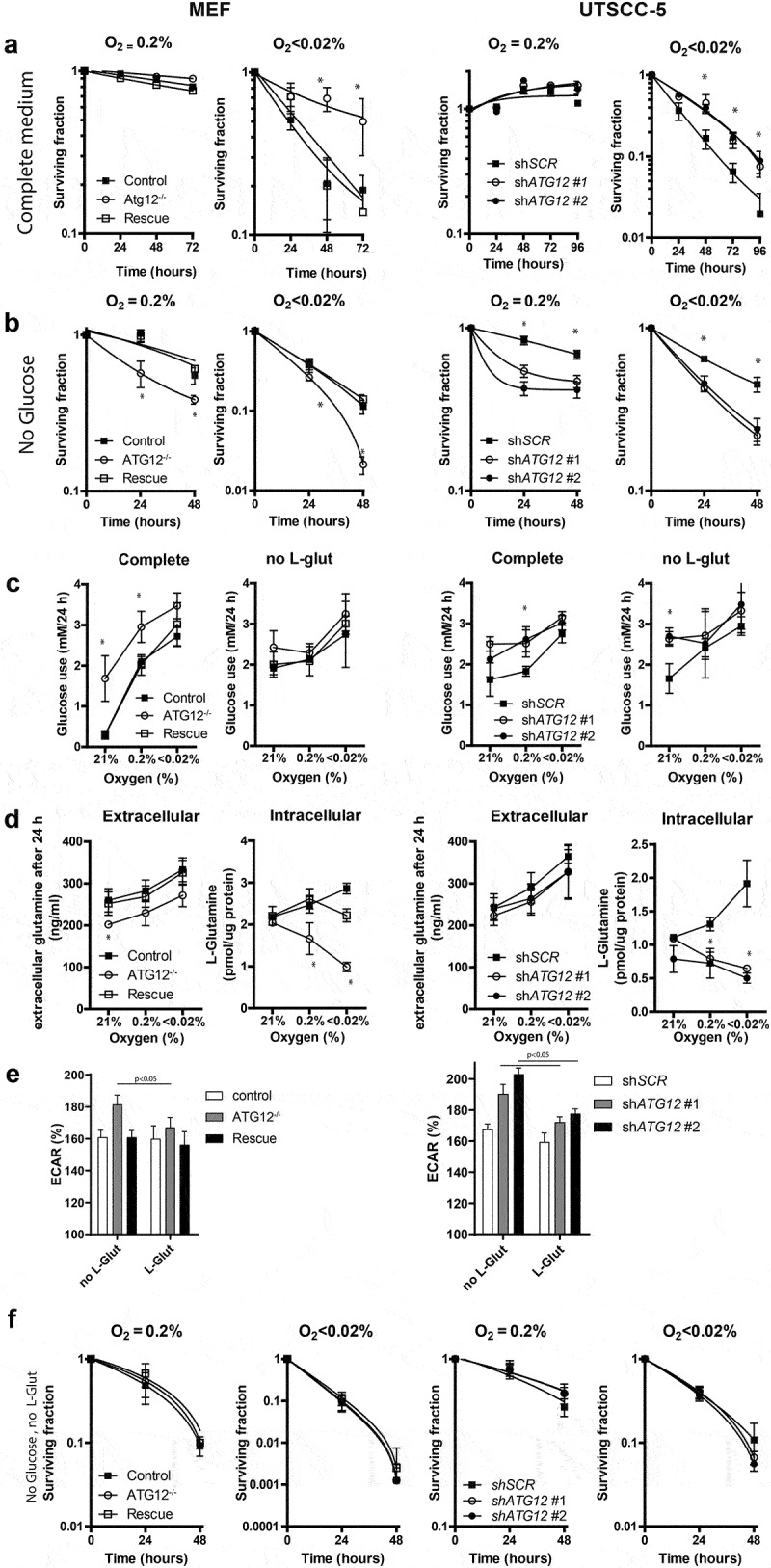
ATG12 is required for glutamine homeostasis and mediates survival during hypoxia in MEFs and UTSCC5 cells. (A) Clonogenic survival of control, *ATG12*^−/−^ and *ATG12*^−/−^ where *ATG12* is re-expressed (rescue) MEF (left panels) or UTSCC5 cells that express control or *ATG12*-targeting shRNA (right panels) after exposure to moderate (O2 = 0.2%) or severe (O2 < 0.02%) hypoxia in complete medium (mean ± SEM, n = 3). (B) Clonogenic survival of MEF (left panels) and UTSCC5 cells after exposure to moderate and severe hypoxia in glucose-depleted medium (mean ± SEM, n = 4). (C) Glucose use by MEF and UTSCC5 cells over 24 h in complete medium (left panels) and in L-glutamine depleted medium (right panels) under ambient oxygen, moderate and severe hypoxia (mean ± SEM, n = 4). (D) Extracellular (left panels) and intracellular (right panels) after 24 h incubation under ambient oxygen, moderate or severe hypoxia (mean ± SEM, n = 4). (E) basal glycolysis as determined ECAR (Seahorse) of MEF (left panel) and UTSCC5 cells (right panel) in the absence or presence of 2 mM L-glutamine (mean ± SEM, n = 4). (F) Clonogenic survival of MEF and UTSCC5 cells after exposure to moderate or severe hypoxia in the absence of glucose and L-glutamine in culture medium during exposure (mean ± SEM, n = 4) * indicates p < 0.05.

Previously we showed that genetic and pharmacological inhibition of autophagy sensitizes cells to hypoxia [10,28,29]. In line, the absence of tumor hypoxia in ATG12^low^ tumors in patients (Figure 1F) suggests that ATG12-deficient cells are less resistant to hypoxia than ATG12 proficient cells. As ATG12 is also involved in other cell death and homeostatic processes [17,30], we tested if ATG12 is required for survival during hypoxia. Surprisingly, exposure of ATG12-deficient MEF, UTSCC5 (Figure 5A) and HT29 (Fig S3A) cells in complete cell culture medium to moderate (0.2% O_2_, 16 h) or to severe hypoxia (<0.02% O_2_,16 h) did not result in increased cell killing as determined by clonogenic survival assays. In contrast, during severe hypoxia, ATG12-deficiency even resulted in increased survival. Tumor hypoxia is the result of limited oxygen delivery via the blood and oxygen-usage by cells at closer proximity to the delivering blood vessel. Besides hampered oxygen delivery, the poorly developed vasculature results in limited glucose delivery to the same areas and may vary from 10 mM to 0.5 mM [31,32], resulting in a larger dependence on other carbon sources.

Cells were therefore exposed to hypoxia in the absence of glucose in the culture medium. Strikingly, the survival advantage of ATG12-deficient MEFs, UTSCC5 and HT29 cells during severe hypoxia in complete medium is completely abrogated when exposed to hypoxia in the absence of glucose (Figure 5B and Fig S3B, right panels). Also increased cell killing was observed during moderate hypoxia (Figure 5B and Fig S3B, left panels). Clonogenic survival under ambient air in the absence of glucose displayed no survival differences (Fig S4A). Interestingly, elevated glucose use during hypoxia (MEFs and UTSCC5) and ambient oxygen (MEFs) was observed in ATG12-deficient cells (Figure 5C, left panels).

Glutamine is the most abundant amino acid in the human blood. It can be liberated from protein complexes through autophagy-dependent degradation, is a source of carbon to support energy generation, metabolite generation and fatty acid biosynthesis [33]. In line, in pancreatic ductal carcinoma, autophagy deficiency results in increased glutamine consumption and lowering of intracellular glutamine [34]. During hypoxia, glutamine is increasingly utilized as alternative carbon source, is required for lipogenesis and nucleotide biosynthesis and supports tumor growth [33,35]. L-glutamine depletion from culture medium resulted in increased glucose metabolism in control cells during ambient air exposure in MEF, but no increase was observed in ATG12-deficient cells. In UTSCC5 cells, no differences were observed for glucose use during hypoxia. During ambient oxygen, ATG12-deficient cells displayed elevated glucose use compared to control cells (Figure 5C, right panels).

In the absence of glucose, glutamine is utilized by cells as alternative carbon source. We therefore explored glutamine metabolism in ATG12-deficient cells. Extracellular levels of L-glutamine are elevated after 24 h hypoxia exposure in MEF, UTSCC5 (Figure 5D), HT29 (Fig S3C), suggesting reduced metabolism or increased liberation from protein complexes and increased efflux from cells. Surprisingly, exposure to hypoxia results in increased or stabilized *intracellular* L-glutamine levels in control cells, but a rapid reduction in ATG12-deficient cells (Figure 5D and Fig S3C), suggesting that intracellular L-glutamine is dependent on L-glutamine supplementation from media but also from intracellular sources. This is further illustrated by the enhanced reduction in intracellular L-glutamine, when the exogenous source is removed during 16-h exposure to hypoxia (Fig S4B). Metabolic profiling indicated that in the absence of L-glutamine, ATG12-deficient cells display increased basal glycolysis, as determined by Seahorse measurements (Figure 5E, left bars). Supplementation of exogenous L-glutamine to the assay medium resulted in reduced basal glycolysis in ATG12-deficient cells. No changes were observed in ATG12-proficient cells, suggesting that for metabolic processes, ATG12-deficient cells are more responsive and reliant on exogenous glutamine (Figure 5E, right bars). In addition, ATG12-deficient cells increase their maximum glycolytic capacity upon glutamine addition, suggesting that these cells are able to upscale their glycolysis even further when glutamine is added to the medium and the mitochondrial coupled ATP-production is inhibited and indicate elevated dependence on glutamine for their metabolic activity (Fig S5). In support of the redundant roles of glucose and L-glutamine as carbon source during hypoxia, we observed that depleting both glucose and L-glutamine resulted in abrogation of the survival disadvantage of ATG12-deficient cells (Figure 5F and Fig S3D). In these circumstances, intracellular L-glutamine levels are fully depleted and below limit of detection in both ATG12 pro- and deficient cells (data not shown).

**Figure 6.**
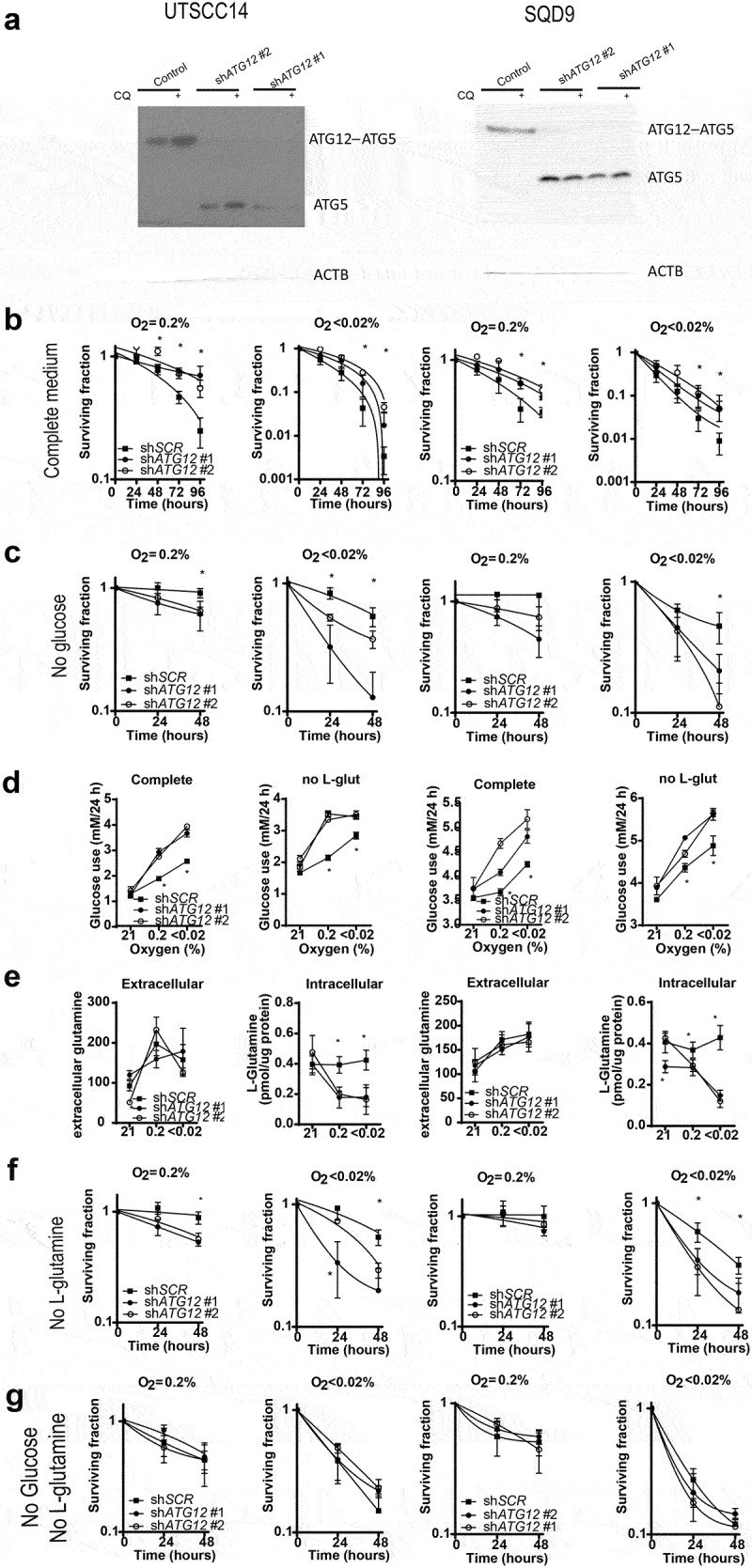
ATG12 deficiency results in disruption of glutamine homeostasis in UTSCC14 and SQD9 cells. (A) immunoblot of UTSCC14 and SQD9 cells expressing a doxycycline inducible control shRNA or *ATG12*-targeting shRNA. Clonogenic survival of SCR (n = 6) shRNA #1 (n = 3) or sh*ATG12* #2 (n = 3) expressing UTSCC14 (left panels) or SQD9 (right panels) after exposure to moderate (O_2_ = 0.2%) or severe (O2 < 0.02%) hypoxia in (B) complete or (C) glucose deprived culture medium (mean ± SEM). (D) Glucose use over 24 h in complete medium (left panels) and in L-glutamine depleted medium (right panels) under ambient oxygen, moderate and severe hypoxia (mean ± SD). (E) Extracellular (left panels) and intracellular (right panels) L-glutamine of SCR (n = 7), sh*ATG12* #1(n = 3) and sh*ATG12* #2 (n = 4) cells after 24 h incubation under ambient oxygen, moderate or severe hypoxia (mean ± SD). Clonogenic survival of shSCR (n = 6) sh*ATG12* #1 (n = 3) or sh*ATG12* #2 (n = 3) expressing UTSCC14 (left panels) or SQD9 (right panels) after exposure to moderate (O_2_ = 0.2%) or severe (O2 < 0.02%) hypoxia in (F) L-glutamine- deprived or (G) glucose- and L-glutamine-deprived culture medium. * indicates p < 0.05.

The observed results were further validated in two additional HNSCC cell lines. Similar to the other cell lines, ATG12 deficiency resulted in a survival advantage in UTSCC14 and SQD9 cells (Figure 6A a nd B). This effect could be abrogated by hypoxia exposure in the absence of glucose, resulting in enhanced cell killing of ATG12-deficient cells (Figure 6C). In UTSCC14 and SQD9 cells, no differences in glucose use were observed in complete medium during ambient oxygen exposure. During hypoxic exposure, glucose use increased in ATG12-deficient cells independent of exogenous L-glutamine availability (Figure 6D). Comparable to UTSCC5 cells, extracellular levels of L-glutamine increase after 24-h hypoxia exposure (Figure 6E) and ATG12 deficiency rapidly results in reduced levels of intracellular glutamine in UTSCC14 and SQD9 cells (Figure 6E, right panels). In the absence of glucose or exogenous glutamine, intracellular L-glutamine levels are even further reduced, suggesting preferred or elevated glutamine metabolism during hypoxia. Comparable to nutrient rich conditions, in glucose- or exogenous L-glutamine depleted media, intracellular L-glutamine levels are reduced in ATG12-deficient cells (Fig S6). Together these results confirm that intracellular L-glutamine is dependent on L-glutamine supplementation from media but also from intracellular sources.

We observed that glucose depletion sensitized ATG12-deficienct cells to hypoxia (Figure 5B and) and our data suggest that this effect is mediated through the failure to maintain adequate intracellular L-glutamine levels. To further determine if the survival advantage of control cells over ATG12-deficient cells is mediated through L-glutamine availability, cellular survival was determined in the absence of exogenous L-glutamine. Exposure to moderate hypoxia resulted in enhanced cell killing of ATG12-deficient UTSCC14 cells but not of ATG12-deficient SQD9 cells (Figure 6F). Yet, exposure to severe hypoxia resulted in enhanced cell killing of ATG12-deficient UTSCC14 and SQD9 (Figure 6F), confirming the increased dependence of ATG12-deficient cells on external L-glutamine sources. The requirement for an external carbon source during hypoxia, is further illustrated by the observation that depleting both glucose and L-glutamine results comparable survival of control and ATG12-deficient cells (Figure 6G). Both in control and ATG12-deficient cells, depletion of both carbon sources results in L-glutamine levels below detection limit (data not shown).

**Figure 7.**
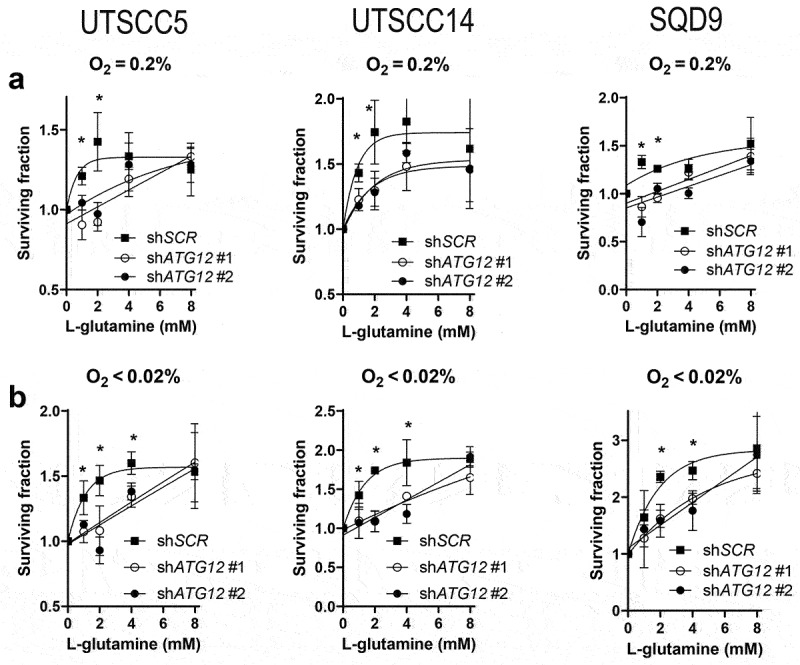
Supplementing ATG12-deficient cells with L-glutamine restores cellular survival during hypoxia. Clonogenic survival of control or ATG12-deficient UTSCC5 (left), UTSCC14 (middle) and SQD9 (right) cells after exposure to moderate (A) or severe (B) hypoxia in the absence of glucose and supplemented with 0, 1, 2, 4 or 8 mM L-glutamine (mean ± SEM, n = 3). * indicates p < 0.05.

To further explore the increased dependence of ATG12-deficient cells on exogenous L-glutamine accessibility, cellular survival after 48 h was assessed in UTSCC5, UTSCC14 and SQD9 cells exposed to hypoxia in glucose deprived medium supplemented with 0,1,2,4 or 8 mM L-glutamine. Supplementing control cells with 1 or 2 mM L-glutamine rapidly results in increased cellular survival and reaches a maximum at 4 mM L-glutamine (Figure 7A and B). In contrast, ATG12-deficient cells are less responsive to L-glutamine levels up to 2 mM, as observed before (Figure 5B). Nevertheless, at 4 mM and at 8 mM survival of ATG12-deficient cells becomes comparable to control cells. These data indicate that, intrinsically, ATG12-deficient cells can compensate for low glucose levels by utilization of L-glutamine, yet at very high concentrations. This observation suggests that multiple mechanisms that may comprise of liberation, processing, import or metabolism is subject to ATG12 expression.

**Figure 8.**
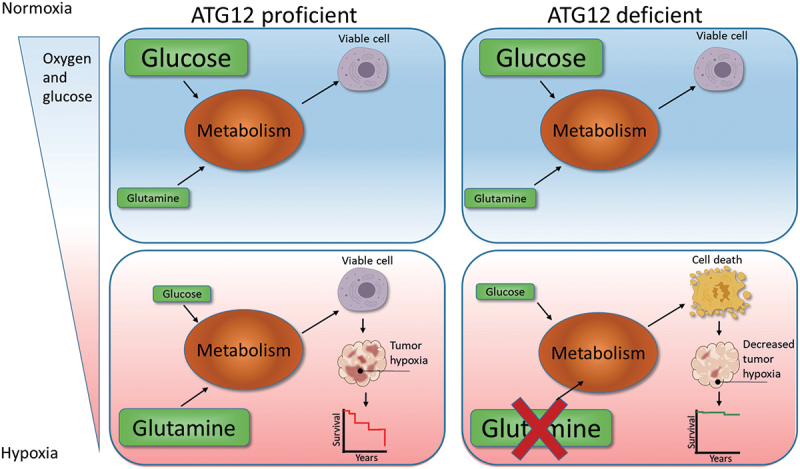
Model for ATG12 dependence during hypoxia. During normal oxygenation and sufficient glucose abundance, glucose is used as the preferred carbon source. Areas that receive insufficient oxygen are also hampered in glucose delivery and are therefore more reliant on glutamine metabolism as carbon source. ATG12 proficient cells are capable of maintaining high intracellular glutamine levels and maintain a viable radiotherapy-resistant hypoxic fraction. ATG12-deficient cells cannot maintain high intracellular glutamine during hypoxia, resulting in hypoxic cell death, decreased tumor hypoxia and improved prognosis after therapy.

In short (Figure 8), hypoxia, resulting from decreased delivery of oxygen through the vasculature, is accompanied by reduced availability of glucose and L-glutamine. Oxygen and glucose delivery cannot, or only to a very limited extent, be compensated by endogenous sources. During hypoxia intracellular L-glutamine levels increase and may act as alternative metabolic substrates. Our data indicate that ATG12-deficient tumors are incapable of maintaining intracellular L-glutamine levels and therefore cannot support a significant hypoxic fraction (Figure 1F), which results in improved treatment response in HNSCC (Figures 2, 3, and 4). Although the majority of the manuscript is focused on HNSCC, comparable observations were done in MEF and a colorectal cell line (HT29), suggesting that the proposed mechanism is applicable to other normal cells and other tumor types as well. Nevertheless, this will require further investigation.

## Discussion

In this study, we show that expression of the essential autophagy gene ATG12 is lost in a subpopulation of HPV-negative HNSCC. Interestingly, patients with ATG12-deficient and autophagy-defective tumors display increased local and loco-regional control and improved overall survival in stage III and IV of the disease. The fact that the survival advantage is not limited to HNSCC alone, but is also observed in ovarian, breast, pancreas and several renal cancers, indicates a general characteristic is changed in these tumors. In solid cancers, tumor hypoxia is an important component that limits treatment efficacy [1]. A meta-analysis indicated that the degree of tumor hypoxia is the most significant factor explaining variability in survival in head and neck cancers^9^. Hence, a number of markers, such as tumor hypoxia and tumor cell proliferation, have been described to predict tumor control. In line with the essential role of autophagy in hypoxic cells survival, analysis of the TME revealed that ATG12-deficient tumors lack the presence of hypoxic regions. The loss of ATG12 expression is associated with an allelic deletion and potential loss of flanked genes, resulting in an effect that may not be related to ATG12 itself. Interestingly, according to the TCGA HNSCC database, mRNA abundance of the genomically closest genes *CDO1* (cysteine dioxygenase type 1), *DDX43* (DEAD-box helicase 43) and *RNU2-49P* (RNA, U2 small nuclear 49, pseudogene) display no correlation with ATG12 expression (data not shown). Only *AP3S1* (adaptor related protein complex 3 subunit sigma 1) displays a modest correlation (r = 0.56), indicating that other regulatory mechanisms are suppressing efficient ATG12 expression. In contrast to patient tumors, screening of >20 cancer cell lines, including 8 HNSCC, available in our laboratory indicated that all *in vitro* cell lines express ATG12 mRNA and protein (not shown). This suggests that the suppressing factor is dependent on (microenvironmental) factors that are absent in 2D cell cultures or that ATG12-expression provides an advantage for establishment or proliferation of *in vitro* cultures. In line with ATG12-specific effects, controlled *in vivo* studies with specific ATG12 knockdown xenografts repeated the observations in clinical specimens. Despite reduced vasculature, reduced vessel perfusion and thus reduced oxygenation in ATG12-deficient tumors, comparable hypoxia was observed. Nevertheless, this was associated with a dramatic increase in tumor necrosis in ATG12-deficient tumors and indicates reduced hypoxia tolerance of ATG12-deficient cells. We observed that tolerance to hypoxia of ATG12-deficient cells is related the severity of hypoxia and external access to carbon sources such as glucose and L-glutamine (figure 5 and Figure 6). Pimonidazole, used in our experiments to mark hypoxia, labels cells below 10 mmHg (~1.3% O_2_), but cannot discriminate between cells exposed to different oxygen tensions below 10 mmHg. The detected hypoxic fractions may therefore represent fractions with distinct severities in hypoxia. After delivery of a large dose of irradiation, regrowth of the tumor is dependent on the number of surviving hypoxic cells. Yet, despite comparable hypoxic fractions, ATG12-deficient tumors proved to be very radiosensitive (Figure 4H a nd I). Radiosensitivity increases as the oxygen tension increases sharply from anoxia to ∼10 mmHg [36]. Small differences in severity of hypoxia below 10 mmHg may therefore result in large differences in tumor regrowth.

These data illustrate the importance of ATG12 in maintaining cell viability in hypoxic areas. In line with the clinical data, the intratumoral changes improved response to radiotherapy. Previously, it was shown that single nucleotide polymorphisms (rs26537) in ATG12 is associated with increased expression of ATG12 and increased risk of HNSCC [37]. The level of ATG12 expression within the tumor therefore seems to be a decisive characteristic in HNSCC tumorigenesis and treatment efficacy. Whether this is also applicable to other cancer types, remains to be investigated.

Core proteins associated with autophagy are in general not subjected to a high degree of somatic mutations in cancer and expression patterns considered to be protected from alterations [38]. Nevertheless, we observed that a large subpopulation of HNSCC patients displayed severely reduced or absence of ATG12 mRNA and protein expression. The loss of autophagy results in genomic instability and oncogenic stimulation that increases cancer initiation [39–41]. As such, genetically engineered mouse models (GEMM) deficient in autophagy are prone to development of tumors (reviewed in [42]). Paradoxically, autophagy supports growth of advanced cancers. As also observed in the current study, this is, at least in part, mediated through maintaining viability of hypoxic cells within the tumor microenvironment [10,29]. Furthermore, studies using GEMM showed that *Atg7*-deficient KRAS^G12D^-driven lung cancer is reduced in proliferation capacity and trigger an immune response [43]. Comparable observations have been made in GEMM for breast, pancreatic, colorectal, prostate cancer, melanoma and glioblastoma (reviewed in [42]). In line with these observations, Yang *et al*. showed that inducible inhibition of autophagy by expressing and enzyme-inactive form of Atg4B in as “wild-type” established tumor, resulted in growth inhibition [44]. Tumors that regrew were selected for loss of expression of the enzyme-inactive Atg4B mutant and confirm the proliferation and survival advantage of autophagy-proficient advanced tumors. Combined, these models indicate that autophagy inactivation suppresses tumor growth, immune evasion, survival and malignancy and phenotypically may be considered a different subclass of tumors. In line with a distinct subclass, we observed enhanced response to therapy and a favorable outcome for patients with ATG12-deficient tumors.

The necrotic phenotype of ATG12 knockdown xenografts indicates that, in line with patient data, these tumor cells are less resistant to hypoxia. Surprisingly, *in vitro* survival studies in culture medium with glucose and glutamine levels suggested that ATG12 is not required for survival during hypoxia per se. Typically, autophagy inhibition sensitizes cells to hypoxia resulting in decreased cell survival [10]. Nevertheless, ATG12 has pro-apoptotic properties by interacting with anti-apoptotic BCL2 family members to promote apoptosis by cytochrome c release [17].

This suggests that at this conversion point between autophagy and apoptosis, the role of ATG12 is probably more pro-apoptotic. These findings are in line with previous studies that show that silencing ATG12 improves cell survival after stimulation with apoptosis inducers [45] and indicate that, in nutrient rich conditions, the role of ATG12 in pro-death mechanisms is stronger than pro-survival mechanisms like autophagy.

However, in hypoxic regions, also the delivery of glucose is impaired and the limited available glucose is converted into secretory lactate. As an alternative carbon-source, hypoxic cells can exert to glutamine metabolism, resulting in increased glutamine metabolism. Besides glutamine as building block for protein synthesis, metabolic changes result in biosynthesis of non-essential amino acids, nucleotides and fatty acids [33]. Hypoxia rapidly leads to elevated activation of autophagy. High autophagy activity provides the cell with energy and liberation of essential building blocks such as sugars, lipids, and amino acids. This is also illustrated by the increased availability of non-protein associated extra- and intracellular glutamine during hypoxia (Figure 5D). In line, we and others observed that autophagy deficiency results in lowering of intracellular glutamine [34]. We propose that in hypoxic tumor regions, oxygen and glucose are inefficiently delivered and cells become, at least in part, reliant on glutamine metabolism.

During hypoxic exposure, autophagy-proficient cells are capable of maintaining higher intracellular L-glutamine levels in nutrient rich conditions (Figure 5D and Figure 6C), but also during conditions when the exogenous source is removed (Fig S6). Depletion of both glucose and L-glutamine from culture media, results in non-measurable intracellular L-glutamine levels in ATG12 pro- and deficient cells (data not shown) and illustrates the importance of having at least one carbon source to maintain intracellular L-glutamine homeostasis.

What the exact mechanism is of lowered intracellular L-glutamine levels in ATG12-deficient cells is, will require further research. For example, autophagy deficiency in these cells may lead to reduced L-glutamine liberation from protein complexes and results in lowering of intracellular L-glutamine levels. Alternatively, changes in processing and/or metabolism could result in enhanced L-glutamine turnover in ATG12-deficient cells. Although the importance of L-glutamine in inducing autophagy has been well established [46], whether (failure in) activation of autophagy results in changes in L-glutamine turnover or controls a feedback mechanism remains unclear. Additionally, although ATG proteins play a central role in the formation of autophagy-related structures, they also control localization and function of membrane proteins [12] and ion channels and transporters [47]. Currently, it is unclear if glutamine transporters are dependent on autophagy for localization, expression or activity.

Many cancers are dependent on excessive glutamine metabolism for growth and survival. Hence in almost all cancer cell culture media L-glutamine is supplemented. Our results suggest that the disturbance in L-glutamine homeostasis in ATG12-deficient cells results in increased cell killing during hypoxia. It is tempting to speculate that ATG12 proficient cells, through maintaining higher intracellular levels via autophagy mediated recycling, display increased survival. If autophagy would indeed be essential for hypoxia survival via maintaining glutamine levels, then autophagy intact cells should survive better in the absence of both glucose and L-glutamine. Nevertheless, we observed that in the absence of both, no survival differences are observed between ATG12 pro- and deficient cells during hypoxia (Figure 5F and Figure 6G). This intriguing observation suggests that (i) autophagy dependent liberation is insufficient to compensate for loss of exogenous sources, (ii) L-glutamine metabolism exceeds liberation or (iii) that the role of autophagy mediated L-glutamine liberation is limited in mediating cellular survival during hypoxia. Interestingly, we observed that when both carbon sources are removed, the levels of intracellular L-glutamine are below detection limit in autophagy pro- and deficient cells (data not shown).

That the function of ATG12 in maintaining intracellular L-glutamine homeostasis and cellular survival is complex, is further illustrated by rescue experiments (Figure 7). Although in ATG12 proficient cells 2 mM L-glutamine is sufficient to induce survival, in ATG12-deficient cells this requires 4 or 8 mM for complete rescue in glucose deprived conditions. These results suggest that other, or additional mechanisms than autophagy-dependent liberation from protein complexes, may be controlled by ATG12 expression. What the exact mechanism is, remains to be investigated.

In short, we observed that ATG12-deficient cells are sensitive to depletion of either glucose or L-glutamine from culture media. Depletion of either carbon source results in increased cell death during hypoxia exposure. We propose that the limited availability of these exogenous carbon sources in the tumor microenvironment results in killing of ATG12-deficient cells during hypoxia, resulting in low tumor hypoxia and improved outcome after therapy (Figure 8).

In contrast to intracellular levels, we observed that cells increase extracellular L-glutamine levels during exposure to hypoxia, irrespective of ATG12 expression (Figure 5D and Figure 6E). This could be the net effect of several processes. For example, besides autophagy, hypoxia results in the accumulation of misfolded proteins that are subject to proteasomal degradation. Several reports indicate that the initial degradation response is mediated through ER associated protein degradation (ERAD) and that, once ERAD is saturated, autophagy will be activated to maintain homeostasis [48]. Additionally, hypoxia results in cell death and dying cells may release aminoacids, proteins and degrading enzymes into the cell culture supernatant. Thirdly, hypoxic conditions may stimulate the conversion of glutamate to glutamine [49] and result in elevated L-glutamine secretion. Lastly, intracellular and extracellular L-glutamine levels are subject to import:export ratios and changes therein. Microarray analyses on a series of timepoints of cells that are exposed to hypoxia indicate that several glutamine transporters (SLC7A5, SLC7A6, SLC38A1 and SLC38A2) display at least 4-fold differences in mRNA expression within 24 h after hypoxia exposure (data not shown). SLC7A5 acts as an efflux pump of glutamine from the cell and may contribute to the rise in extracellular glutamine. Glutamine efflux regulates MTOR, translation and autophagy, and is essential for the import of essential amino acids [50].

Intracellular L-glutamine is essential for several important (homeostatic) processes within the cell (e.g. fuel, aminoacid import, protein production, carbon source) and maintaining sufficient intracellular glutamine levels is critical. It is not surprising that cells have several independent mechanisms to achieve this. Intracellular L-glutamine levels are determined by a combination of import/efflux, liberation from protein complexes, release from storages, and conversion from glutamate. Our data indicates that during hypoxia, ATG12 is essential in maintaining intracellular homeostasis and is required to compensate the increased glutamine efflux from cells. Most likely, all mechanisms that support intracellular L-glutamine availability are required in combined effort to maintain sufficient levels and interference in any of these mechanisms could, potentially, yield the same phenotype. Here, we have identified autophagy as a critical mechanism for maintenance of sufficient intracellular L-glutamine levels.

The classical HNSCC is traditionally driven by excessive tobacco and alcohol consumption and is considered a distinct disease from the human papilloma virus driven oropharyngeal SCC [51]. The HPV-orginated tumors are characterized by a good prognosis leading to a new staging system and discussions in de-escalating treatment intensity to limit toxicity [52]. Here, we identified a subtype of (HPV-negative) HNSCC with a very favorable prognosis. As tumor hypoxia is the largest determinant in treatment-efficacy variability in survival in head and neck cancers [3] many endogenous markers such as HIF1A, CA9, SLC2A1 and others, have been investigated. However, expression of endogenous markers at the site of biopsy does not always comprise the temporo-spatial fluctuations of hypoxia due to its heterogeneous and dynamic characteristics. In contrast to endogenous markers of hypoxia, which are *dependent* on tumor hypoxia, ATG12 expression seems to predict *if* a tumor has the capacity to support tumor hypoxia. Similar to HPV, ATG12 therefore has the potential to serve as a predictive marker for local and loco/regional control in HNSCC.

## Materials and methods

### Patient cohorts

RADPLAT, n = 86 [53] and ARCON, n = 17 [22]) cohorts were combined and were used for analyses of Figure 2. ATG12 expression was determined by qPCR and normalized to 18S expression. Both cohorts were approved by the respective local ethics committee. Data was sorted and split in quartiles; 1^st^ (low ATG12, 28 patients) and 2^nd^-4^th^ quartile (high ATG12 expression, 75 patients). The majority of patients were male (64% in ATG12^low^ vs 73% in ATG12^high^) with a comparable median age (56.6 vs 56.7). All patients were treated with a combination of radiotherapy (70 Gy, 2 Gy fractions) with cisplatin or accelerated radiotherapy (64–68 Gy, 2 Gy fractions) with and without carbogen breathing (Table S1). Local control and loco-regional control were used to evaluate outcome.

Patient cohorts of *The Cancer Genome Atlas* (Genomic data commons (GDC) processed cohorts) were accessed via a portal of the Santa Cruz University of California (https://xenabrowser.net/). Patient cohorts were filtered for availability of ATG12 expression data, tumor vs control tissue and tumor type. For HNSCC, HPV positive tumors were excluded from the analysis. Data was sorted on ATG12 expression and split in quartiles (1^st^ (low ATG12) and 2^nd^-4^th^ quartile (high ATG12 expression). Copy number was also extracted from the database (patient characteristics are listed in Table S2). Other cohorts (Ovarian cancer, infiltrating ductal breast cancer, ductal pancreatic cancer, renal clear cell carcinoma, renal papillary cell carcinoma and kidney chromophobe cancer) were split based on median ATG12 expression (patient characteristics are listed in Table S3). Outcome was evaluated by overall survival. Curve comparison was done using the log-rank test (Graphpad prism software).

### Quantitative PCR

RNA extraction was done using Nucleospin RNA kit (Macherey-Nagel, 740.955.250) and cDNA was prepared using I-script (Bio-Rad, 1,708,891). Patient RNA was extracted using TRI reagent (Sigma Aldrich, T9424) according to the manufacturers manual Real-time PCR was done using a Bio-Rad T100 Thermal Cycler. mRNA abundance was measured using SYBR green Lo-ROX (GC Biotech, QT625-20). Abundance of each gene was normalized to *RNA 18S ribosomal N1-N5 (18S)*. Primers are listed in Table 1.

**Table 1.** Primers used for qPCR determination.

Target	Forward	reverse
*ATG12* set 1	AGGTCTGTAGTCGCGGAGAA	CGGGAACACCAAGTTTCACT
*ATG12* set 2	CCCAGACCAAGAAGTTGGAA	TGATGCTTGTGGCAAGAGAC
*ATG12* set 3	CTTACGGATGTCTCCCCAGA	CGAACCATCCAAGGACTCAT
*LC3B*	AACGGGCTGTGTGAGAAAAC	AGTGAGGACTTTGGGTGTGG
*VEGF*	TCCGGGCTCGGTGATTTA	GACTCCGGCGGAAGCAT
*ATG5*	GACCTTCAGTGGTCCGGTAA	GCAAGCCAGACAGGAAAAAG
*RNA18S* N1-N5	AGT GCCC TGC CCT TTG TAC ACA	GAT CCG AGG GCC TCA CTA AAC

### Immunohistochemistry and image processing

For patient biopsy analysis, frozen acetone fixed cross sections were stained using ATG12- (D88H11; Cell Signaling Technology, 4180) and pimonidazole (Chemicon, HP-1000 mg)-specific antibodies. Tumor biopsies without or only few (<1%) ATG12-positive cells were grouped as ATG12^low^. Pimonidazole and 9F1 rat monoclonal antibody to mouse endothelium (Radboud University Nijmegen, Dept. of Pathology) were stained as described previously [54]. Hypoxia, necrosis, endothelium and perfusion (Hoechst) was quantified by computerized digital image processing system using fluorescent microscope with a computer-controlled motorized stepping stage, equipped with a high-res intensified solid-state camera (Axioskop; Zeis). Necrosis was examined morphologically by H&E staining. Hypoxic fraction and (perfused) vascular densities are indicated as fraction of the total viable tumor area.

### Cell culture

HT29 (colorectal adenocarcinoma; American Type Culture Collection, HTB-38), UTSCC5 (established from a tumor from the tongue, kindly provided by Dr. Reidar Grenman, University of Turku, Finland) [55], SQD9 (squamous cell carcinoma of the larynx, kindly provided by Prof. A Begg, the Netherlands Cancer Institute) and UTSCC14 (squamous cell carcinoma of the tongue, kindly provided by Dr. Reidar Grenman) were cultured in DMEM (Sigma, D6429-24) and DMEM supplemented with 0.1 mM non-essential amino acids (Sigma, M7145-100ML) supplemented with 10% FCS. Hypoxia exposure (16–20 h overnight) was done using a modular atmosphere-controlled system (Don Whitley Scientific, H85). Doxycycline-inducible knockdown of ATG12 was achieved through lentiviral transduction and expression of shRNA from pTRIPZ backbones TGATGAAGTCAATGAGTCC (#1) and AAACAACTGTTCTGAGGCC (#2). Knockdown was induced 6 days prior to experiment. MEFs were isolated as described elsewhere [56].

### Tumor models

To study the role of ATG12 on tumor growth and its impact on radiotherapy, doxycycline inducible sh*ATG12*-UTSCC5 cells were injected subcutaneously in the flanks of NMRI^nu/nu^ mice (1.0E6 cells/50 ul matrigel [Corning, 354,230). Doxycycline (Sigma Aldrich, 24,390) was administered via the drinking water (2 g/L, 5% sucrose). Tumor size was assessed by caliper measurements [54]. Animals were injected with BrdUrd (30 mg/ kg; Sigma Aldrich, 59–14-3) and pimonidazole (60 mg/ kg; Hypoxyprobe, HP-1000 mg) intraperitoneally 1 h prior to killing. For irradiation experiments, after reaching 150 mm^3^ in tumor size, animals were anesthetized (ketamine and xylazine) and tumors were irradiated with a single, tumor-specific, dose of 10 Gy (16 MeV, Varian Truebeam® machine running version 2.5 using ServiceMode). The animals were positioned on the couchtop and irradiated with electrons using a field of 25 cm x 25 cm, source skin distance of 100 cm whereby the bodies were shielded with lead (thickness 2 cm). All animal experiments were conducted in accordance with national guidelines and approved by the animal ethics committee Maastricht University.

### Clonogenic survival assays

Knockdown was induced by doxycycline 7 days before experiments. Cells were seeded and exposed to moderate (0.2%O_2_) or severe (O_2_ < 0.02%) hypoxia using a modular atmosphere-controlled system. Cells were seeded in complete medium and left to adhere for 6 h, then the medium was changed and incubated in complete DMEM (Thermo Fisher, 41,966–029), DMEM w/o glucose (Thermo Fisher, 11,966,025) or DMEM w/o glucose and glutamine (Sigma, A1443001). As similar setup was used for the glutamine spike in experiments. Cells were seeded in complete medium and left to adhere for 6 h. Then the medium was changed to DMEM w/o glucose and glutamine or supplemented with 1,2,4 or 8 mM L-glutamine (Westburg, LO BE17-605E) and directly exposed to hypoxia for 48 h. After exposure, medium was changed without doxycycline and incubated for ± 10 days to allow colony formation. Colonies were color-fixed (0.4% methylene blue in 70% EtOH; Sigma Aldrich, M4159) and counted manually. Colonies existing of at least 50 cells were counted.

### Glucose and glutamine determination

Glucose was determined in culture medium according to the manufacturers’ instruction (Biosentec, 010). Extracellular L-glutamine was determined in culture medium after incubation using a glutamine assay kit (Abcam, ab197011). To assess intracellular glutamine, cells were lysed, and glutamine content was corrected for total protein.

### Extracellular acidification rate (ECAR)

Extracellular acidification rate of MEFs (20,000 cells/well), UTSCC5 (20,000 cells/well) and HT29 (35,000 cells/well) was measured using the XF^e^96 extracellular flux analyzer (Agilent Technologies, Santa Clara, CA, USA). Cells were seeded in DMEM containing L-glutamine (Gibco, 11,995,065), supplemented with MEM Non-essential amine acids solution acids (Sigma, M7145-100ML), and 10% FCS (Sigma-Aldrich, F7524) and incubated overnight in an incubator containing 5% CO_2_ at 37°C.

The effect of L-glutamine on ECAR was determined after replacing the medium to Seahorse XF Base Medium (Agilent Technologies, 103,334–100) with or without 2 mM L-glutamine and 1 h equilibration in a CO_2_-free incubator at 37°C. First baseline ECAR was determined followed by glucose addition (final conc 10 mM). Shift in ECAR, corrected for baseline were considered as basal glycolysis. Data were corrected for protein content of the wells after cell lysis.

### Immunoblotting

Cells were lysed in RIPA buffer (50 mM HEPES-KOH, pH 7.5, 150 mM KCl, 1 mM EDTA, 2 mM mercaptoethanol, 0.2% Tween-29 (Sigma Aldrich, R0278) with protease inhibitor (Roche, 4,693,124,001) and sonicated 3 × 5 s at 10 MHz. Samples were separated by SDS-PAGE and transferred to PVDF membranes (VWR, 10,600,023) and probed with primary antibodies ATG5 (1:1000, D5F5U; Cell Signaling Technology, 12,994) ATG12 (1:1000, D88H11; cell signaling Technology, 4180) LC3B (1:1000; MBL, PM036) ACTB/β-actin (1:200.000; MP Biomedicals, 691,001). Primary antibodies were visualized using HRP-linked secondary antibodies (anti-rabbit, anti-mouse, 1:2000; Cell Signaling Technology, 7076). Super Signal West Pico chemiluminescent substrate (Thermo Scientific, 34,579) was used for detection.

### Statistical analyses

Statistical analyses were performed using graphpad prism software. Student’s t test was used for single comparisons. Corrections for multiple testing was done using within repeated measures ANOVA with bonferroni post-hoc testing. Chi square was used to test for differences in distribution of clinical populations. Differences in survival were analyzed through log rank (Mantel-Cox) testing. Differences were considered statistically different when p < 0.05.

## Supplementary Material

Supplemental Material
