# Supplementary material for: ATG12 deficiency results in intracellular glutamine depletion, abrogation of tumor hypoxia and a favorable prognosis in cancer
Source: Autophagy. 2021 Dec 14;18(8):1898–914. doi: 10.1080/15548627.2021.2008690 (PMC9450974; doi:10.1080/15548627.2021.2008690)
Supplement: Supplemental Material [file KAUP_A_2008690_SM0589.zip › supplementary/Supplementary figures R4-done.docx]

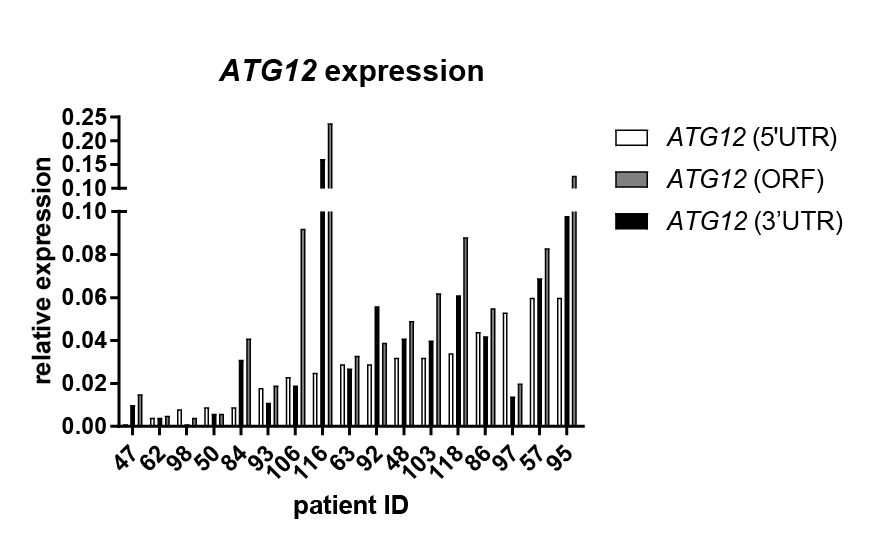


**Figure S1.** The identical pattern of the 3 independent primer pairs indicate loss of the full *ATG12* transcript expression.


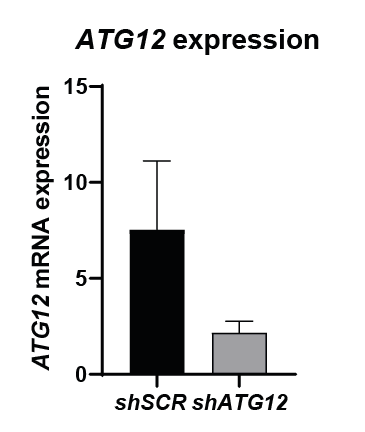


**Figure S2.** *ATG12* mRNA expression normalized to *RNA18S* at the end of the tumor

xenograft experiment of figure 4B and C, indicating that ATG12 knockdown is

maintained over the whole experiment (mean +/- stdev, *shSCR* n=5, sh*ATG12* n=6).


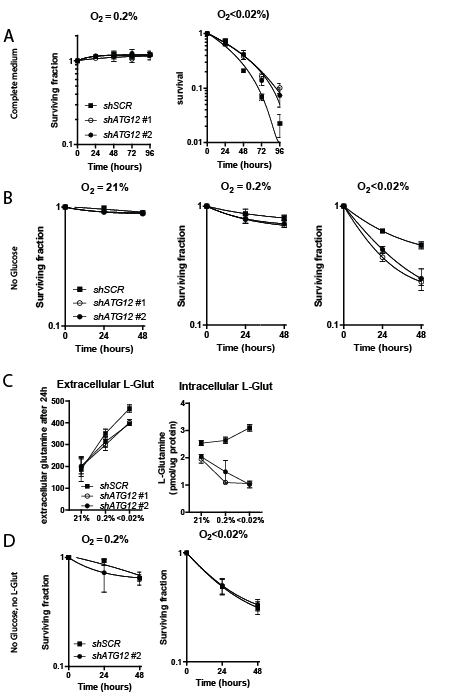


**Figure S3.** ATG12 is required for glutamine homeostasis and mediates survival during hypoxia in HT29 cells. (**A**) Clonogenic survival of HT29 cells that express control or *ATG12* targeting shRNA after exposure to moderate (O2=0.2%) or severe (O2<0.02%) hypoxia in complete medium (mean +/- SEM, n=3). (**B**) Clonogenic survival of HT29 cells after exposure to moderate and severe hypoxia in glucose-depleted medium (mean +/- SEM, n=4). (**C**) Extracellular and intracellular glutamine after 24 h incubation under ambient oxygen, moderate or severe hypoxia (mean +/- SEM, n=4). (**D**) Clonogenic survival HT29 cells after exposure to moderate or severe hypoxia in the absence of glucose and L-glutamine in culture medium during exposure (mean +/- SEM, n=4).


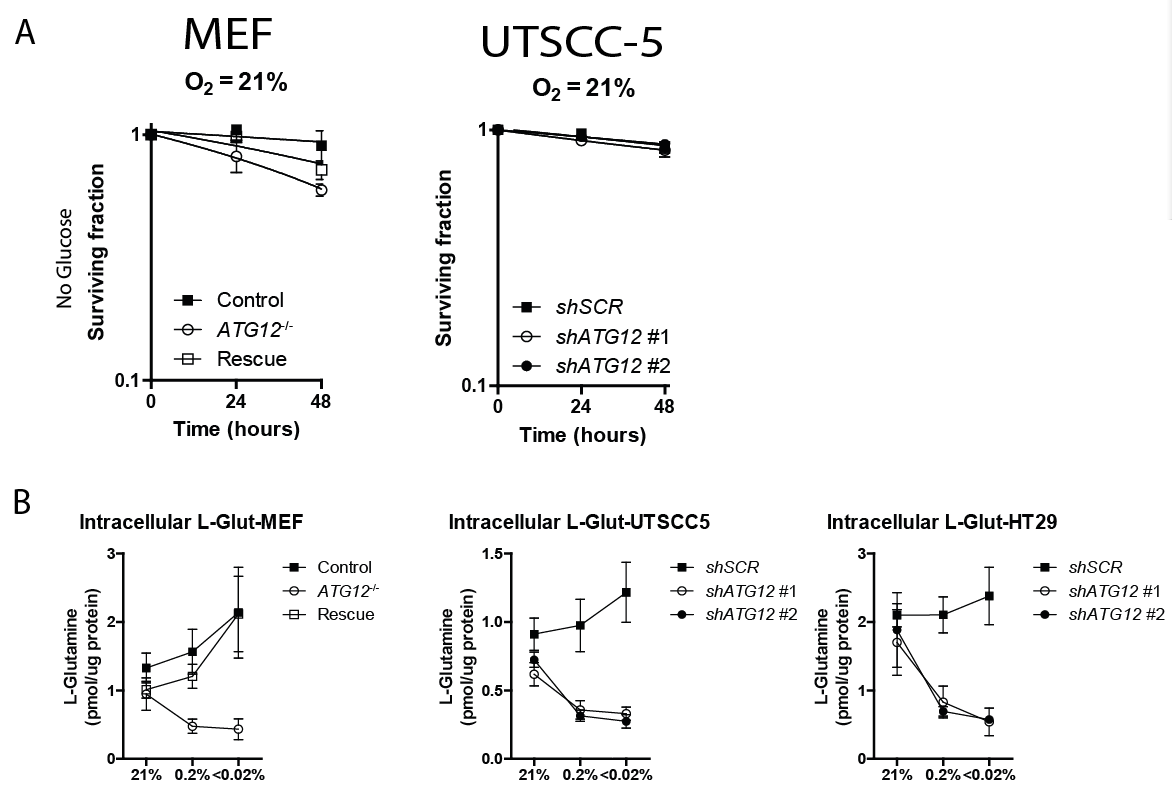


**Figure S4.** *ATG12-*deficiency results in intracellular L-glutamine depletion. (**A**) Clonogenic survival of MEF (left panels) and UTSCC5 cells after exposure to ambient air in glucose-depleted medium (mean +/- SEM, n=4). (**B**) Intracellular glutamine after 24-h incubation under ambient oxygen, moderate or severe hypoxia in the absence of supplemented extracellular L-glutamine (mean +/- SEM, n=4).


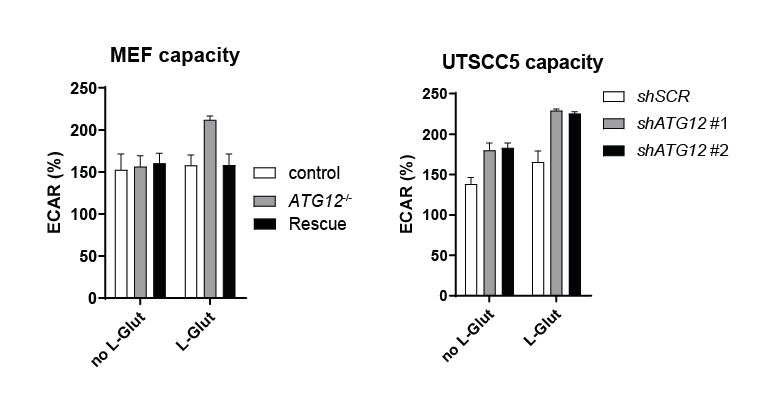


**Figure S5.** Maximum glycolytic capacity as determined by ECAR (Seahorse) of MEF (left panel) and UTSCC5 cells (right panel) in the absence or presence of 2 mM L-glutamine (mean +/- SEM, n=4).


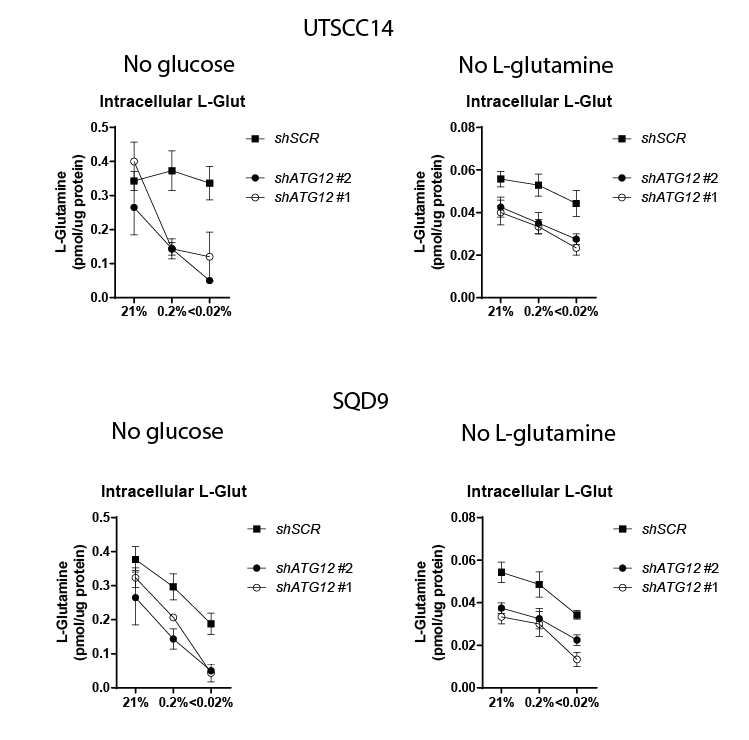


**Figure S6.** Intracellular glutamine after 24-h incubation under ambient oxygen, moderate or severe hypoxia in the absence of glucose (left panels) or in the absence of exogenous L-glutamine (right panels) (mean +/- SEM, n=3).
