# Supplementary material for: ATG12 deficiency results in intracellular glutamine depletion, abrogation of tumor hypoxia and a favorable prognosis in cancer
Source: Autophagy. 2021 Dec 14;18(8):1898–914. doi: 10.1080/15548627.2021.2008690 (PMC9450974; doi:10.1080/15548627.2021.2008690)
Supplement: Supplemental Material [file KAUP_A_2008690_SM0589.zip › supplementary/Supplementary tables R4 done.docx]

| **Table S1.** Patient characteristics. |  |  |  |
| --- | --- | --- | --- |
|  | **Total** | **ATG12 Low** | **ATG12 High** |
| **Number of patients** | *103 (100%)* | *28 (27%)* | *75 (73%)* |
| **male** | *73 (71%)* | *18 (64%)* | *55 (73%)* |
| **female** | *30 (29%)* | *10 (36%)* | *20 (27%)* |
| **Age (mean)** | *57.2* | *59.4* | *57.2* |
| **range** | *29.1-79.1* | *42.7-77.3* | *29.1-79.1* |
| **median** | *56.3* | *56.6* | *56.7* |
| **Histological site** |  |  |  |
| **oropharynx** | *54 (52%)* | *15 (54%)* | *39 (52%)* |
| **hypopharynx** | *23 (22%)* | *4 (14%)* | *19 (25%)* |
| **oral cavity** | *13 (13%)* | *4 (14%)* | *9 (12%)* |
| **larynx** | *12 (12%)* | *5 (18%)* | *7 (9%)* |
| **other** | *1 (1%)* | *0 (0%)* | *1 (1%)* |
| **Tumour stage** |  |  |  |
| **1** | *6 (6%)* | *2 (7%)* | *4 (5%)* |
| **2** | *7 (7%)* | *1 (4%)* | *6 (8%)* |
| **3** | *33 (32%)* | *9 (32%)* | *24 (32%* |
| **4** | *56 (54%)* | *14 (50%)* | *42 (56%)* |
| **Treatment** |  |  |  |
| **RT*+ cisplatin (IV)** | *23 (22%)* | *5 (18%)* | *18 (24%)* |
| **RT*+ cisplatin (IA)** | *33 (32%)* | *6 (21%)* | *27 (30%)* |
| **RT*+ cisplatin (LD)** | *30 (29%)* | *11 (39%)* | *19 (25%)* |
| **RT **(accelerated)** | *2 (2%)* | *1 (4%)* | *1 (1%)* |
| **RT** (ARCON/CN)** | *9 (9%)* | *3 (11%)* | *6 (8%)* |
| **RT** (ARCON)** | *1 (1%)* | *- ( -%)* | *1 (1%)* |
| **RT** + Surgery** | *3 (3%)* | *2 (7%)* | *1 (1%)* |
| **RT**(conventional)** | *2 (2%)* | *- ( -%)* | *2 (3%)* |

* All patients were treated with 70 Gy in 2 Gy daily fractions. five times per week [55]. 100 mg/m2 cisplatin was administered IV three times during radiotherapy or 150 mg/m2 given IA four times during radiotherapy. whereas the others received daily LD of cisplatin (20 × 6 mg/m2).
** Accelerated radiotherapy was given to a total dose of 64–68 Gy in 2-Gy fractions within 36–38 days. This was combined with carbogen breathing during irradiation and administration of nicotinamide (60–80 mg/kg) 1–1.5 h before irradiation.

**Table S2.** TCGA patient characteristics.

|  |  | | |  | | |
| --- | --- | --- | --- | --- | --- | --- |
|  | **all stages** | | | **stage 3 and 4** | | |
|  | **total** | **ATG12 low** | **ATG12 High** | **total** | **ATG12 low** | **ATG12 High** |
| **No of patients** | *479 (100%)* | *119 (25%)* | *359 (75%)* | *374 (100%)* | *95 (25%)* | *279 (75%)* |
| **male** | *347 (72%)* | *92 (77%)* | *255 (71%)* | *284 (76%)* | *78 (82%)* | *206 (74%)* |
| **female** | *132 (28%)* | *27 (23%)* | *104 (29%)* | *90 (24%)* | *17 (18%)* | *73 (26%)* |
| **Age** |  |  |  |  |  |  |
| **mean** | *61.3* | *60.9* | *61.5* | *60.9* | *59.9* | *61.1* |
| **range** | *19-90* | *26-87* | *19-90* | *29-87* | *26-82* | *19-90* |
| **Histological site** |  |  |  |  |  |  |
| **larynx** | *113 (24%)* | *39 (33%)* | *74 (21%)* | *98 (26%)* | *34 (36%)* | *64 (23%)* |
| **hypopharynx** | *9 (2%)* | *4 (3%)* | *5 (1%)* | *9 (2%)* | *4 (4%)* | *5 (2%)* |
| **oropharynx** | *45 (9%)* | *8 (7%)* | *37 (10%)* | *36 (10%)* | *6 (6%)* | *30 (11%)* |
| **oral cavity** | *312 (65%)* | *69 (58%)* | *243 (68%)* | *232 (62%)* | *52 (55%)* | *180 (65%)* |
|  |  |  |  |  |  |  |
| **Stage** |  |  |  |  |  |  |
| **1** | *25 (5)%* | *5 (4)%* | *20 (6)%* | *- (-%)* | *- (-%)* | *- (-%)* |
| **2** | *79 (16)%* | *19 (16)%* | *60 (17)%* | *- (-%)* | *- (-%)* | *- (-%)* |
| **3** | *89 (19)%* | *22 (18)%* | *67 (19)%* | *89 (24)%* | *22 (23)%* | *67 (24)%* |
| **4** | *286 (60)%* | *74 (62)%* | *212 (59)%* | *286 (76)%* | *74 (78)%* | *212 (76)%* |
|  |  |  |  |  |  |  |
| **Treatment** |  |  |  |  |  |  |
| **radiation** | *140 (29%)* | *37 (31%)* | *103 (30%)* | *115 (67%)* | *46 (48%)* | *69 (24%)* |
| **Targeted molecular treatment** | *4 (0.8%)* | *- (-%)* | *4 (1%)* | *4 (1%)* | *4 (4%)* | *- (-%)* |
| **radiation +TMT** | *105 (22%)* | *24 (20%)* | *81 (22%)* | *98 (26%)* | *22 (23%)* | *76 (27%)* |
| **unknown** | *229 (48%)* | *58 (48%)* | *171 (48%)* | *157 (42%)* | *23 (24%)* | *134 (48%)* |

**Table S3A.** Patient characteristics ovarian cancer.

|  | *ATG12* low |  |  | *ATG12* high |  | total |
| --- | --- | --- | --- | --- | --- | --- |
|  |  |  |  |  |  |  |
| Number of patients | 187 |  |  | 188 |  | 375 |
|  |  |  |  |  |  |  |
| **Age at diagnosis** |  |  |  |  |  |  |
|  |  |  |  |  |  |  |
| median (year) | 59.9 |  |  | 59.6 |  |  |
| mean | 60.7 |  |  | 59.6 |  |  |
| Range | 35.0 to 87.5 |  |  | 30.5 to 87.6 |  |  |
|  |  |  |  |  |  |  |
|  |  |  |  |  |  |  |
| **Clinical stage** |  | % |  |  | % |  |
| stage 1 | 0 | 0.0 |  | 0 | 0.0 |  |
| stage 1a | 0 | 0.0 |  | 0 | 0.0 |  |
| stage 1b | 0 | 0.0 |  | 0 | 0.0 |  |
| stage 1c | 1 | 0.5 |  | 0 | 0.0 |  |
| stage 2 | 0 | 0.0 |  | 0 | 0.0 |  |
| stage 2a | 0 | 0.0 |  | 1 | 0.5 |  |
| stage 2b | 1 | 0.5 |  | 2 | 1.1 |  |
| stage 2c | 9 | 4.8 |  | 8 | 4.3 |  |
| stage 3a | 2 | 1.1 |  | 5 | 2.7 |  |
| stage 3b | 8 | 4.3 |  | 5 | 2.7 |  |
| stage 3c | 136 | 72.7 |  | 138 | 73.4 |  |
| stage 4 | 28 | 15.0 |  | 29 | 15.4 |  |
| N/A | 2 | 1.1 |  | 0 | 0.0 |  |

**Table S3B.** Patient characteristics infiltrating ductal breast.

| \|  \| *ATG12* low \|  \|  \| *ATG12* high \|  \| \| --- \| --- \| --- \| --- \| --- \| --- \| \| Number of patients \| 432 \|  \|  \| 442 \|  \| \|  \|  \|  \|  \|  \|  \| \| **Age at diagnosis** \|  \|  \|  \|  \|  \| \| median (year) \| 56.6 \|  \|  \| 59.3 \|  \| \| mean \| 57.3 \|  \|  \| 58.5 \|  \| \| Range \| 27.0 to 90.1 \|  \|  \| 26.6 to 90.1 \|  \| \|  \|  \|  \|  \|  \|  \| \| **Tumor stage** \|  \| **%** \|  \|  \| **%** \| \| stage 1 \| 34 \| 7.9 \|  \| 46 \| 10.4 \| \| stage 1a \| 33 \| 7.6 \|  \| 38 \| 8.6 \| \| stage 1b \| 2 \| 0.5 \|  \| 3 \| 0.7 \| \| stage 2 \| 1 \| 0.2 \|  \| 3 \| 0.7 \| \| stage 2a \| 155 \| 35.9 \|  \| 141 \| 31.9 \| \| stage 2b \| 112 \| 25.9 \|  \| 99 \| 22.4 \| \| stage 3a \| 50 \| 11.6 \|  \| 70 \| 15.8 \| \| stage 3b \| 8 \| 1.9 \|  \| 12 \| 2.7 \| \| stage 3c \| 15 \| 3.5 \|  \| 16 \| 3.6 \| \| stage 4 \| 11 \| 2.5 \|  \| 7 \| 1.6 \| \| N/A \| 11 \| 2.5 \|  \| 7 \| 1.6 \| \|  \|  \|  \|  \|  \|  \| \| **ER status** \|  \|  \|  \|  \|  \| \| Positive \| 46 \| 10.6 \|  \| 40 \| 9.0 \| \| Negative/Undertermined \| 386 \| 89.4 \|  \| 402 \| 91.0 \| \|  \|  \|  \|  \|  \|  \| \| **Radiotherapy** \|  \|  \|  \|  \|  \| \| Yes \| 221 \| 51.2 \|  \| 100 \| 22.6 \| \| no \| 161 \| 37.3 \|  \| 174 \| 39.4 \| \| N/A \| 50 \| 11.6 \|  \| 168 \| 38.0 \| |  |  |  |  |  |
| --- | --- | --- | --- | --- | --- | --- | --- | --- | --- | --- | --- | --- | --- | --- | --- | --- | --- | --- | --- | --- | --- | --- | --- | --- | --- | --- | --- | --- | --- | --- | --- | --- | --- | --- | --- | --- | --- | --- | --- | --- | --- | --- | --- | --- | --- | --- | --- | --- | --- | --- | --- | --- | --- | --- | --- | --- | --- | --- | --- | --- | --- | --- | --- | --- | --- | --- | --- | --- | --- | --- | --- | --- | --- | --- | --- | --- | --- | --- | --- | --- | --- | --- | --- | --- | --- | --- | --- | --- | --- | --- | --- | --- | --- | --- | --- | --- | --- | --- | --- | --- | --- | --- | --- | --- | --- | --- | --- | --- | --- | --- | --- | --- | --- | --- | --- | --- | --- | --- | --- | --- | --- | --- | --- | --- | --- | --- | --- | --- | --- | --- | --- | --- | --- | --- | --- | --- | --- | --- | --- | --- | --- | --- | --- | --- | --- | --- | --- | --- | --- | --- | --- | --- | --- | --- | --- | --- | --- | --- | --- | --- | --- | --- | --- | --- | --- | --- | --- | --- | --- | --- | --- | --- | --- | --- | --- | --- | --- | --- | --- |

**Table S3C.** Ductal pancreatic cancer.

|  | *ATG12* low |  |  | *ATG12* high |  |
| --- | --- | --- | --- | --- | --- |
| Number of patients | 71 |  |  | 72 |  |
|  |  |  |  |  |  |
| **Age at diagnosis** |  |  |  |  |  |
| median (year) | 65.9 |  |  | 65.9 |  |
| mean | 64.9 |  |  | 65.5 |  |
| Range | 36.0 to 82.4 |  |  | 41.4 to 86 |  |
|  |  |  |  |  |  |
| **Tumor stage** |  | **%** |  |  | **%** |
| stage i | 0.0 | 0.0 |  | 0.0 | 0.0 |
| stage 1a | 1.0 | 1.4 |  | 2.0 | 2.8 |
| stage 1b | 4.0 | 5.6 |  | 6.0 | 8.3 |
| stage 2 | 0.0 | 0.0 |  | 0.0 | 0.0 |
| stage 2a | 11.0 | 15.5 |  | 12.0 | 16.7 |
| stage 2b | 52.0 | 73.2 |  | 48.0 | 66.7 |
| stage 3 | 1.0 | 1.4 |  | 2.0 | 2.8 |
| stage 3a | 0.0 | 0.0 |  | 0.0 | 0.0 |
| stage 3b | 0.0 | 0.0 |  | 0.0 | 0.0 |
| stage 4 | 2.0 | 2.8 |  | 1.0 | 1.4 |
| N/A | 0.0 | 0.0 |  | 1.0 | 1.4 |
|  |  |  |  |  |  |
| **Gender** |  |  |  |  |  |
| male | 32.0 | 45.1 |  | 44.0 | 61.1 |
| female | 39.0 | 54.9 |  | 28.0 | 38.9 |
|  |  |  |  |  |  |
| **Surgery** |  |  |  |  |  |
| wipple | 56.0 | 78.9 |  | 57.0 | 79.2 |
| distal pancreatectomy | 8.0 | 11.3 |  | 8.0 | 11.1 |
| other | 7.0 | 9.9 |  | 6.0 | 8.5 |

**Table S3D.** Renal clear cell carcinoma.

|  | *ATG12* low |  |  | *ATG12* high |  |
| --- | --- | --- | --- | --- | --- |
|  |  |  |  |  |  |
| Number of patients | 292 |  |  | 296 |  |
|  |  |  |  |  |  |
| **Age at diagnosis** |  |  |  |  |  |
| median (year) | 60.7 |  |  | 61.8 |  |
| mean | 60.5 |  |  | 61.8 |  |
| Range | 29.3 to 86.5 |  |  | 26.6 to 88.7 |  |
|  |  |  |  |  |  |
| **Tumor stage** |  | **%** |  |  | **%** |
| stage 1 | 153 | 52.4 |  | 125 | 42.2 |
| stage 2 | 31 | 10.6 |  | 36 | 12.2 |
| stage 3 | 54 | 18.5 |  | 84 | 28.4 |
| stage 4 | 52 | 17.8 |  | 50 | 16.9 |
| N/A | 2 | 0.7 |  | 1 | 0.3 |
|  |  |  |  |  |  |
| **Gender** |  |  |  |  |  |
| male | 178 | 61.0 |  | 214 | 72.1 |
| female | 114 | 39.0 |  | 83 | 27.9 |

**Table S3E.** Kidney papillary cell carcinoma.

|  | *ATG12* low |  |  | *ATG12* high | |
| --- | --- | --- | --- | --- | --- |
|  |  |  |  |  |  |
| Number of patients | 148 |  |  | 140 |  |
|  |  |  |  |  |  |
| **Age at diagnosis** |  |  |  |  |  |
| Median (year) | 63.6 |  |  | 60.5 |  |
| Mean | 62.8 |  |  | 61.3 |  |
| Range | 35.3 to 88.1 |  |  | 28.3 to 85.5 | |
|  |  |  |  |  |  |
| **Tumor stage** |  | % |  |  | % |
| stage i | 98 | 66.2 |  | 74 | 52.9 |
| stage 2 | 14 | 9.5 |  | 7 | 5.0 |
| stage 3 | 21 | 14.2 |  | 30 | 21.4 |
| stage 4 | 4 | 2.7 |  | 11 | 7.9 |
| N/A | 11 | 7.4 |  | 18 | 12.9 |

**Table S3F.** Kidney chromophobe.

|  | *ATG12* low | |  | *ATG12* high |  |
| --- | --- | --- | --- | --- | --- |
|  |  |  |  |  |  |
| Number of patients | 38 |  |  | 27 |  |
|  |  |  |  |  |  |
| **Age at diagnosis** |  |  |  |  |  |
| median (year) | 47.7 |  |  | 51.6 |  |
| mean | 52.1 |  |  | 52.9 |  |
| Range | 29.2 to 75.3 | |  | 18 to 86.6 |  |
|  |  |  |  |  |  |
| **Tumor stage** |  | % |  |  | % |
| stage i | 12 | 31.6 |  | 8 | 30.8 |
| stage 2 | 15 | 39.5 |  | 11 | 42.3 |
| stage 3 | 10 | 26.3 |  | 4 | 15.4 |
| stage 4 | 1 | 2.6 |  | 3 | 11.5 |
|  |  |  |  |  |  |
| **Gender** |  | % |  |  |  |
| male | 23 | 60.5 |  | 16 | 59.3 |
| female | 15 | 39.5 |  | 11 | 40.7 |
