# Supplementary material for: ATG12 deficiency results in intracellular glutamine depletion, abrogation of tumor hypoxia and a favorable prognosis in cancer
Source: Autophagy. 2021 Dec 14;18(8):1898–914. doi: 10.1080/15548627.2021.2008690 (PMC9450974; doi:10.1080/15548627.2021.2008690)
Supplement: Supplemental Material [file KAUP_A_2008690_SM0589.zip › supplementary/Supplementary_KAUP-2020-0457R3_accept.docx]

**Sharma et al: AUTOPHAGY MODULATE CELL FATE DECISIONS DURING LINEAGE COMMITMENT**

**Supplementary figures**

**Figure S1**. Autophagy is induced during DE differentiation. (**A**) Schematics of the DE differentiation process. The upper panel illustrates the normal process of differentiation of hiPSCs into DE, and the middle panel shows the base media, time course and small-molecule additions during the differentiation process. The lower panel indicates the key markers expressed at different stages of differentiation into DE. (**B**) Pluripotency characterization of AG27 seeded as single cells. The cells were grown for 24 h and fixed, followed by immunofluorescence microscopy with POU5F1/OCT4, SOX2 and NANOG antibodies. Scale bar: 50 µm. (**C**) Representative images of the morphology of AG27 hiPSCs plated as single pluripotent cells at different stages of differentiation to DE. Representative images (n=3) taken at the indicated time points post induction of differentiation are shown. Scale bar: 100 µm. (**D**) Immunofluorescence analysis of FOXA2 and SOX17 protein expression at the DE differentiation endpoint (48 h) (n=3). Nuclei were stained with DAPI. Scale bar: 100 µm. (**E-J**) AG27 hiPSCs were treated with CHIR to induce DE differentiation, collected at the indicated time points and processed for RT-qPCR using specific TaqMan primers against (E) differentiation stage specific genes; *MIXL1 (*marker of primitive streak), *FOXA2 (*mesendoderm and DE marker) and *SOX17 (*DE marker) and (F-J) autophagy-related genes; (F) *MAP1LC3B*, (G) *SQSTM1*, (H) *WDFY3*, (I) *ATG5*, (J) *ATG7*. The relative expression levels were normalized to that at 0 h and are presented as the mean of three independent experiments (n=3). Error bars represent SD. ** P<0.01, *** P<0.001. (**K**) Autophagy kinetics during DE formation. AG27 hiPSCs were lysed in RIPA buffer at the indicated time points until DE formation (confirmed by RTqPCR and fluorescence microscopy) and were processed for western blotting using PIK3C3, phospho-RPS6KB/p70S6K and GABARAP antibodies. The blots are representative of three independent experiments. ACTB was used as a loading control. (**L**) Representative images of the morphology of the human ESC line H1 at different stages of differentiation into DE. The images were taken at 4, 8, 12, 24 and 48 h post induction of differentiation. Scale bar: 50 µm. (**M**) Changes in the levels of autophagy related proteins during the differentiation of the human ESC line, H1, to definitive endoderm (DE). The H1 cells were lysed in RIPA buffer at 0 and 48 h and the following proteins, WDFY3, p-ULK1, SOX17 and MAP1LC3, were analyzed by western blotting. The blots are representative of three independent experiments. ACTB was used as a loading control.

** Figure S2.** Characterization of cardiomyocyte and neuroectoderm differentiation. (**A**) Immunofluorescence analysis of mCherry-EGFP (dt-LC3, uninduced) AG27 hiPSCs for the expression of pluripotency markers SOX2, POU5F1 and NANOG. Nuclei were stained with DAPI. Scale bar: 50 µm. (**B**) Immunofluorescence analysis of ACTN1 in beating cardiomyocytes differentiated from AG27 hiPSCs at day 17 (n=3). Nuclei were stained with DAPI. Scale bar: 10 µm. (**C**) Characterization of the expression profile of key genes during cardiomyocyte formation. hiPSCs differentiated into beating cardiomyocytes were collected at the day beating started, and the mRNA levels of developmentally relevant markers of cardiomyocyte, *NKX2.5* (NK2 homeobox 5) and *TNNT2* (troponin T2, cardiac type) were tested by RT-qPCR using TaqMan analysis. The relative expression levels were normalized to that at 0 h and are presented as the mean of three independent experiments (n=3); error bars represent SD. ** P<0.01, *** P<0.001. (**D**) Characterization of neuroectoderm formation. Cells were collected on day 6 during neuroectoderm differentiation and processed for western blotting using PAX6 and NES antibodies. ACTB was used as a loading control (n = 3). (**E**) *PAX6* expression analysis during neuroectoderm differentiation. The cells were collected every alternate day during neuroectoderm formation and processed for RTqPCR using TaqMan probe against *PAX6*. The graph indicates relative transcript levels and is a representation of three independent experiments (n = 3). Error bars represent SD. **P<0.01, ***P<0.001. (**F**) *NES* expression analysis during neuroectoderm differentiation. The cells were collected every alternate day during neuroectoderm formation and processed for RTqPCR using TaqMan probes against *NES*. The graph indicates relative transcript levels and is a representation of three independent experiments (n = 3).

** Figure S3.** Generation and characterization of AG27 *ATG7*^+/-^ hiPSC. (**A**) Schematic overview of the array card used for RTqPCR, containing lyophilized SYBR probes for a panel of genes in a 96 well format. cDNA representing different treatments was applied to the array card for analysis. The different colour codes indicate genes representing the different lineages. (**B**) Schematics of *ATG7^+/-^* hiPSC. The figure shows the guide sequence and the deleted region post CRISPR-CAS9 engineering. The start codon is indicated. (**C**) AG27 iPSCs cells were electroporated with guide RNA for *ATG7*. The tracr-RNA was fluorescently labelled with ATTO dye. Clones were screened using restriction fragment length polymorphism (RFLP) against a *BamH1* site that is lost in the *ATG7^+/-^*hiPSC clones. (**D**) The graph shows quantifications of SOX2 for western blots from Figure 3F. Briefly, cells from wild type (WT) and *ATG7^+/-^* hiPSC were differentiated into neuroectoderm using SMAD inhibitors. Lysate was collected every day until day 6 and processed for western blotting (n = 3). ACTB was used as a loading control. Error bars represent SEM. *P<0.1, **P<0.01. (**E**) Pluripotency confirmation in *ATG7^+/-^* cells. The *ATG7^+/-^* cells were collected and processed for RTqPCR using TaqMan probe against *POU5F1, SOX2* and *NANOG*. The graph indicates relative quantification using AG27 parental cells as control. (**F**) Definitive endoderm formation from *ATG7^+/-^* cells. *ATG7^+/-^* cells were processed for definitive endoderm formation. The protein levels of SOX17 were detected at different time points (0 h, 12 h and 48 h) in WT AG27 and *ATG7^+/-^* cells taken towards endoderm. The graph indicates relative SOX17 levels normalized to 0 h. ACTB was used as loading control. Error bars indicate SEM. (**G**) Rosette formation in *ATG7^+/-^* hiPSC clones. In addition to *ATG7^+/-^*hiPSC clone 48 (Figure 3**G**), clone U and clone 54 were also plated and observed for morphological changes. Arrowheads indicate rosette like structures formed after 7 (clone U) and 10 days (clone 54) respectively. Scale bar: 100 µm.

** Figure S4.** Modulation of autophagy regulates differentiation and SOX2 degradation. (**A**) Effect of rapamycin on the differentiation of AG27 iPSCs into DE. The cells were treated with CHIR to induce DE formation in the absence or presence of the MTOR inhibitor rapamycin. The images were taken 48 h post differentiation induction. The upper panel represents cells at 4x magnification, and the lower panel represents 10x. Arrowheads indicate undifferentiated areas post DE differentiation. Scale bar: 100 µm in 4x and 20 µm in 10x. (**B**) The expression of SOX17 during DE differentiation of *ATG7^+/-^* cells. Cell lysate was made from *ATG7^+/-^* cells directed towards DE differentiation at different time points (0 h, 12 h and 48 h, in triplicates) (as in Fig. 4F). The lysates were analyzed by western blotting using antibodies against SOX17. ACTB was used as loading control. (**C**) Confocal microscopy of pluripotent mCherry-EGFP-tagged POU5F1, SOX2 or NANOG human iPSC lines. Expression was induced with doxycyclin for 24 h, followed by imaging of the cells. DAPI was used to counter stain nuclei. Scale bar: 10 µm. (**D**) SOX2 is not degraded during neuroectoderm formation from human iPSCs. mCherry-EGFP-SOX2 iPSCs were induced with doxycyclin and differentiated to neuroectoderm. The cells were fixed at the indicated days post differentiation to study autophagic flux. DAPI was used to counterstain the nucleus. Scale bar: 5 µm.

** Figure S5.** Continuous expression of mCherry-EGFP-SOX2 brings AG27 hiPSCs out of pluripotency. mCherry-EGFP-SOX2 cells were induced with doxycycline every alternate day. The middle panel represents changes in morphology of these cells at 4x magnification whereas the lower panel represents them at 10x magnification (two representative areas) after 8 days of SOX2 overexpression. Scale bar: 100 µm at 4x and 20 µm at 10x.

**Video SV1.** Characterization of mesoderm formation. The AG27 cells were treated with CHIR and IWP2 as detailed in method section. Beating was observed on day 17.

**Video SV2.** Characterization of mesoderm formation. The AG27 cells were treated as in Video S1. The video was recorded 3 months after beating started in the differentiated cells.
